# NUPR1 contributes to radiation resistance by maintaining ROS homeostasis via AhR/CYP signal axis in hepatocellular carcinoma

**DOI:** 10.1186/s12916-022-02554-3

**Published:** 2022-10-19

**Authors:** Yizhi Zhan, Zhanqiao Zhang, Yuechen Liu, Yuan Fang, Yuwen Xie, Yilin Zheng, Guoxin Li, Li Liang, Yi Ding

**Affiliations:** 1 Department of Pathology, Nanfang Hospital and Basic Medical College, Southern Medical University, Guangzhou, 510515 Guangdong China; 2 Guangdong Province Key Laboratory of Molecular Tumor Pathology, Guangzhou, 510515 Guangdong China; 3 Department of General Surgery and Guangdong Provincial Key Laboratory of Precision Medicine for Gastrointestinal Tumor, Nanfang Hospital, The First School of Clinical Medicine, Southern Medical University, Guangzhou, 510515 Guangdong China; 4 Department of Radiation Oncology, Nanfang Hospital, Southern Medical University, Guangzhou, 510515 Guangdong China

**Keywords:** NUPR1, Reactive oxygen species, Hepatocellular carcinoma, Radioresistance, Oxidative stress, Aryl hydrocarbon receptor

## Abstract

**Background:**

Radiotherapy (RT) is one of the major therapeutic approaches to hepatocellular carcinoma (HCC). Ionizing radiation (IR) inducing the generation of reactive oxygen species (ROS) leads to a promising antitumor effect. However, the dysregulation of the redox system often causes radioresistance and impairs the efficacy of RT. Increasing evidence indicates that nuclear protein 1 (NUPR1) plays a critical role in redox reactions. In this study, we aim to explore the role of NUPR1 in maintaining ROS homeostasis and radioresistance in HCC.

**Methods:**

The radioresistant role of NUPR1 was determined by colony formation assay, comet assay in vitro, and xenograft tumor models in vivo. Probes for ROS, apoptosis assay, and lipid peroxidation assay were used to investigate the functional effect of NUPR1 on ROS homeostasis and oxidative stress. RNA sequencing and co-immunoprecipitation assay were performed to clarify the mechanism of NUPR1 inhibiting the AhR/CYP signal axis. Finally, we analyzed clinical specimens to assess the predictive value of NUPR1 and AhR in the radiotherapeutic efficacy of HCC.

**Results:**

We demonstrated that NUPR1 was upregulated in HCC tissues and verified that NUPR1 increased the radioresistance of HCC in vitro and in vivo. NUPR1 alleviated the generation of ROS and suppressed oxidative stress, including apoptosis and lipid peroxidation by downregulating cytochrome P450 (CYP) upon IR. ROS scavenger N-acetyl-L-cysteine (NAC) and CYP inhibitor alizarin restored the viability of NUPR1-knockdown cells during IR. Mechanistically, the interaction between NUPR1 and aryl hydrocarbon receptor (AhR) promoted the degradation and decreased nuclear translation of AhR via the autophagy-lysosome pathway, followed by being incapable of CYP’s transcription. Furthermore, genetically and pharmacologically activating AhR abrogated the radioresistant role of NUPR1. Clinical data suggested that NUPR1 and AhR could serve as novel biomarkers for predicting the radiation response of HCC.

**Conclusions:**

Our findings revealed the role of NUPR1 in regulating ROS homeostasis and oxidative stress via the AhR/CYP signal axis upon IR. Strategies targeting the NUPR1/AhR/CYP pathway may have important clinical applications for improving the radiotherapeutic efficacy of HCC.

**Supplementary Information:**

The online version contains supplementary material available at 10.1186/s12916-022-02554-3.

## Background

Hepatocellular carcinoma (HCC) represents 85% to 90% of all primary liver cancer with poor prognosis [1]. Unfortunately, most HCC patients are diagnosed at an advanced stage and are ineligible for surgery. Radiotherapy (RT), as a local non-invasive treatment, becomes an important alternative approach for advanced HCC [2, 3]. Nevertheless, the anti-HCC efficacy of RT is often limited in intrinsic radioresistant cells or blunted over time by therapy-induced radioresistance [4–6]. Therefore, the molecular mechanisms that govern the radioresistance of HCC urgently need to be explored.

Ionizing radiation (IR) potently induces massive cell death by triggering various biological signals. Among them, reactive oxygen species (ROS) are the key regulator for IR-induced cytotoxicity [7]. Excessive ROS levels caused by IR can disrupt the electron transport chain complexes in mitochondria and induce oxidative stress by reacting with biological molecules, such as lipids, proteins, and DNA [8]. Previous studies showed that ROS homeostasis mediated by the redox system was associated with radioresistance in several malignancies [9, 10]. Tumors resist IR-induced damage by restricting ROS generation or activating antioxidant systems to scavenge free radicals. In breast cancer, activation of STAT3 and Bcl-2 resulted in a persistent reduction of ROS and remarkable radioresistance [11]. In glioblastoma, 6-phosphogluconate dehydrogenase (6PGD) enhanced the pentose phosphate pathway (PPP) to NADPH to detoxify ROS, thereby promoting the radioresistance of cancer [12]. Based on the crucial role of ROS in IR-induced damage, the key molecules that regulate ROS homeostasis and lead to radioresistance in HCC need to be investigated.

NUPR1 (nuclear protein 1) is primarily identified as a transcriptional cofactor strongly induced by several cellular stress [13, 14]. NUPR1 is widely reported to be upregulated in multiple cancers and involved in many cancer-associated processes, including tumor growth [15], invasiveness [16], apoptosis [17], and autophagy [18]. Increasing evidence indicated that NUPR1 could be activated by intracellular ROS and empower tumor cells to survive upon oxidative stress. The inactivation of NUPR1 triggers ROS overproduction due to mitochondrial failure in pancreatic cancer [19]. In addition, NUPR1 is implicated as a modifier on the expression of a series of antioxidant genes, including heme oxygenase-1 (HO-1) [20] and aurora kinase A (AURKA) [21]. NUPR1 protected cancer cells from ferroptosis, one of oxidative cell death, by participating in iron metabolism [22]. All these studies shed light on the possibility that NUPR1 might regulate ROS in HCC. Herein, we aimed to explore the functional role and potential mechanism of NUPR1 in the radioresistance of HCC.

## Methods

### Cell culture and patient samples

HCC cell lines MHCC-97H, MHCC-97L, QGY-7701, Hep3B, Hep1, and Huh7 were purchased from Shanghai Institutes for Biological Sciences (China) and cultured in high glucose Dulbecco’s modified Eagle’s medium (DMEM, Gibco, USA) supplemented with 10% fetal bovine serum (Gibco, USA) at 37°C, 5% CO_2_. Cells were passed every 2–3 days to maintain logarithmic growth and cultured within 35 generations. The short tandem repeat (STR) analysis was used to verify the identity of cell lines.

The human HCC tissue samples and the benign counterparts used in IHC staining of NUPR1 were obtained from the Department of Pathology at Nanfang Hospital (Guangzhou, China) in 2018. A total of 13 specimens from HCC patients who underwent hepatectomy or ultrasonically guided liver biopsy before RT from 2011 to 2019 were also collected from the Department of Pathology at Nanfang Hospital. The therapeutic response of the tumor was evaluated according to the Modified Response Evaluation Criteria in Solid Tumor (mRECIST) as previously described [6]. The collection of human specimens was approved by the Institute Research Medical Ethics Committee of Nanfang Hospital.

### Plasmid constructs, lentivirus, siRNA, and drugs

Lentivirus containing pLent-NUPR1-RFP-Puro (LV-NUPR1) or empty vector (LV-NC) pLent-RFP-Puro were synthesized by Vigene (Vigene Biology, Shandong, China) and used to infect MHCC-97H and MHCC-97L cells with enhanced infection solution (EIS) (Vigene Biology). Similarly, pLent-GFP-sh-NUPR1-Puro (sh-NUPR1) or its negative control (sh-NC) pLent-GFP-Puro (Vigene Biology) was used to infect QGY-7701 and Hep3B cells. Seventy-two hours after the cells were infected with lentivirus, 5 μg/ml puromycin was added to kill the cells that had not been transfected.

The pcDNA3.1 vector (Vigene Biology) containing the full-length cDNA sequence of AhR and empty pcDNA3.1 vector as negative control were used for transient transfection by Lipofectamine 3000 reagents (Invitrogen, Carlsbad, CA) according to the manufacturer’s instructions. Small interfering RNA (siRNA) against NUPR1 (si-NUPR1) and its negative control (si-NC) were obtained from Genechem (Genechem, Shanghai, China) and transfected into HCC cells using Lipofectamine 3000 reagents. The RNA sequences used for transfection in this study are shown in Additional file 1: Table 1.

ROS inhibitor NAC, NUPR1 inhibitor ZZW-115, an agonist of AhR (6-formylindolo[3,2-b]carbazole, FICZ), and specific antagonist of AhR (CH223191) were obtained from MedChemExpress, LLC (Princeton, NJ). Chloroquine (CQ), bafilomycin A1 (BafA1), MG132, and alizarin were purchased from Selleck Chemicals (Houston, USA).

### Colony formation assay

MHCC-97H/MHCC-97L/Hep3B cells (1000–6000/well) and QGY-7701 cells (250–1000/well) were seeded in 6-well plates and treated with different doses of IR (0, 2, 4, 6 Gy). Three thousand MHCC-97H/MHCC-97L/Hep3B cells and five hundred QGY-7701 cells were pretreated with different drugs following exposure to IR (6 Gy). After being cultured for approximately 2 weeks, cells were fixed in methanol and stained with 0.1% crystal violet. Plating efficiency was calculated as the eligible colonies (with > 50 cells)/the number of seeded cells. The survival fraction of cells was the ratio of the plating efficiency of treated cells to that of control cells. All related data were analyzed in GraphPad Prism 8 software, and survival curves of clone formation assays were plotted by using a single-hit multi-targeted model (*y*=1−(1−exp(−k^*^x))^^^*N*).

### Western blot analysis

Protein was separated by 8–12% SDS-PAGE gels, transferred to PVDF membranes (Millipore), and blocked in 5% BSA for 1 h at room temperature. The membranes were incubated overnight at 4°C with primary antibodies. The sources of antibodies against the following proteins were as follows: NUPR1 (15056-1-AP), CYP1B1 (18505-1-AP), CYP3A4 (18227-1-AP), ARNT (14105-1-AP), HSP90 (60318-1-Ig), β-actin (20536-1-AP), LaminB1 (12987-1-AP), LC3 (14600-1-AP) and GAPDH (10494-1-AP) from Proteintech Group; AhR (A4000), CYP1A1 (A2159), caspase 3 (A19654), PARP (A19596), cleaved PARP (A19612) from ABclonal Technology. γH2AX (Ser139; #80312) and p62 (#8025S) was purchased from Cell Signaling Technology. The membranes were washed in PBST and incubated with HRP-conjugated secondary antibodies at room temperature for 1 h. Protein-antibody complexed were visualized using the enhanced chemiluminescence kit (Thermo Fisher Scientific).

### Co-immunoprecipitation assay

Co-immunoprecipitation (Co-IP) assays were performed using MHCC-97H/MHCC-97L cells with NUPR1 overexpression and QGY-7701/Hep3B cells. The cells were harvested in RIPA lysis buffer (P0013D, Beyotime) with a protease inhibitor cocktail for 30 min on ice. The supernatant was collected by centrifugation at 12,000 × g for 15 min. The protein A/G agarose beads were incubated with antibodies overnight at 4°C while rotating. After washing, the complexes were subjected to western blotting analysis. Antibodies used in the study were anti-Flag (F1804, Sigma-Aldrich), NUPR1 (15056-1-AP, Proteintech), AhR (GTX22770, GeneTex), and AhR (A4000, ABclonal). Mouse IgG (B900620) and secondary antibody HRP-goat anti-rabbit IgG (SA00001-2) were from Proteintech.

### Immunofluorescence staining analysis

Cells were seeded on culture dishes and allowed to grow to 70–80% confluency. Cells were washed with PBS and fixed in 4% paraformaldehyde for 15 min. Cells were permeabilized with 0.5% Triton X-100 in PBS for 10 min and washed again with PBS before being blocked with goat serum for 30 min. The fixed cells were incubated overnight at 4°C with primary antibodies against NUPR1 (ab234696, 1:100, Abcam), AhR (GTX22770, 1:100, GeneTex), ARNT (14105-1-AP, 1:200, Proteintech), and LAMP1 (ab208943, 1:100, Abcam). After that, cells were incubated with Alexa Fluor 488-conjugated or Alexa Fluor 555-conjugated secondary antibodies (1:100, Bioss, Beijing) and then mounted with DAPI.

### RNA isolation and real-time PCR

Total RNA was extracted with TRIzol reagent (Invitrogen) and reverse-transcribed into cDNA using the PrimeScript RT Reagent Kit (RR037A, TaKaRa Bio). Quantitative real-time PCR assays were performed by using TB Green Premix Ex Taq II (RR820A, TaKaRa Bio) through an Applied Biosystems 7500 Fast Real-Time PCR System (Thermo Fisher Scientific). Relative expressions were normalized to the geometric mean of housekeeping gene β-actin and were analyzed by using the 2^-ΔΔCt^ method. The primer sequences were listed in Additional file 1: Table 2.

### RNA sequencing

MHCC-97H cells transfected with NUPR1 overexpression vector or control vector were seeded in 6-well plates and exposed to 8 Gy irradiation. After 24 h, total RNA was isolated and subjected to the construction of RNA-seq libraries. The quality of the RNA libraries was evaluated using the Agilent 2200 TapeStation (Agilent Technologies, USA). Library sequencing was performed on a HiSeq 3000 sequencing platform (Illumina Company, USA) by Guangzhou RiboBio Corp., China.

### ROS and lipid peroxidation assay

Cells were seeded in triplicate in 6-well plates and allowed to grow to 70-80% confluency. The cells were pretreated with or without drugs for 24 h and then irradiated. After irradiation for 24 h, MHCC-97H/MHCC-97L cells transfected with RFP protein were replaced with fresh medium containing 5 μM CM-H2DCFDA (C6827, Thermo Fisher) for ROS measurements. QGY-7701/Hep3B cell lines carrying GFP protein were treated with 5 μM CellROX Deep Red Reagent (C10422, Thermo Fisher) to determine ROS levels. A fresh medium with 5 μM BODIPY 581/591 C11 dye (D3861, Invitrogen) was added to each well for lipid peroxidation measurements. After incubation for 30 min in a humidified incubator (37°C, 5% CO_2_), the cells were washed with PBS, digested with trypsin, and measured by flow cytometry using FACS Canto II cytometer (BD Biosciences). The results were analyzed by Flow Jo 7.6.1 software (Treestar).

### Cell death analysis

Cells were seeded in triplicate in 6-well plates and exposed to 8 Gy of irradiation. After IR, cells were replaced with a fresh medium and cultured for three days. Next, cells were washed with PBS, digested by trypsin solution without EDTA, and resuspended in 500 μL assay buffer containing 5 μM of 7-aminoactinomycin D (7-AAD) (C1053S, Beyotime). After incubation for 15 min at room temperature, cell samples were detected and analyzed by flow cytometry (BD Biosciences).

### Apoptosis analysis

For apoptosis analysis, cells were pretreated with or without drugs for 24 h and exposed to a single dose of radiation (8 Gy) for 48 h. The cells were washed by PBS, trypsinized, and resuspended in 500 μL of FITC-Annexin V or APC-Annexin V solution (KeyGen BioTech, Nanjing). After incubation on ice for 15 min, DAPI was added to a final concentration of 10 μg/mL. The samples were then analyzed by flow cytometry (BD Biosciences).

### In vivo xenograft mouse models

For the establishment of HCC xenografts, 2 × 10^6^ MHCC-97H cells transfected with LV-NC/LV-NUPR1 were suspended in 150 μL of serum-free DMEM containing 50 μL Matrigel and subcutaneously injected into nude mice (male, 4–6 weeks). Tumor volumes were measured using digital vernier calipers and calculated by a standard formula: length × width^2^/2. When tumor volume reached 100 mm^3^, irradiation (8 Gy/day × 2 days) was administered to each xenograft. Mice were divided into 4 groups (*n* = 5/group): control, NUPR1 overexpressing, control plus IR, or NUPR1 overexpression plus IR. 1 × 10^7^ Hepa1-6 cells were subcutaneously injected into C57/BL6 mice (male, 4–6 weeks). When xenografts reached about 200 mm^3^, mice were randomly divided into 4 groups (*n* = 5/group): control, ZZW115, IR, or ZZW115 plus IR. A single dose of IR (10 Gy) was given on the first day, and ZZW115 (1 mg/kg) was concurrently given by intraperitoneal injection for 7 consecutive days. The tumors were measured every 4 days and collected when the biggest reached about 1000 mm^3^.

### Immunohistochemistry (IHC) analysis

In brief, paraffin-embedded tissues were cut into 3 μm sections. Sections were deparaffinized, rehydrated, subjected to antigen retrieval, and treated with 3% hydrogen peroxide to block endogenous peroxidase activity. Antibodies against NUPR1 (15056-1-AP, 1:500, Proteintech), AhR (GTX22770, 1:200, GeneTex), CYP1A1 (13241-1-AP, 1: 200, Proteintech), Ki67 (#9449, 1:800, Cell Signaling), PCNA (A12427, 1:200, ABclonal), γH2AX (#9718, 1:400, Cell Signaling), MDA (ab24066, 1:100, Abcam) were incubated with the sections overnight at 4°C, respectively. After incubation with a secondary antibody, the visualization signal was stained with 3, 3′-diaminobenzidine (DAB) and then counterstained with hematoxylin. We regarded the multiplication of staining intensity and the extent of staining as the final score (0–12). Staining intensity was scored as 0 (negative), 1 (weak), 2 (medium) and 3 (strong). The extent of staining scored was as 0 (0%), 1 (1–25%), 2 (26–50%), 3 (51–75%), and 4 (>75%). The stained tissue sections were reviewed and scored separately by two pathologists blinded to the clinical parameters.

### Comet assay

Comet assay was analyzed using DNA Damage Detection Kit (KeyGen BioTech, Nanjing) according to the manufacturer’s instructions. Briefly, transfected cells were irradiated at a dose of 8 Gy. The following day, cells were collected and suspended in PBS containing 1% low-melting agarose and layered onto adhesive microscope slides previously covered with 0.5% normal-melting agarose. The cells were dipped in a specific lysed buffer at 4°C for 2 h. Then, the DNA was uncoiled and unwound in an alkalescent electrophoresis buffer for 30 min. Electrophoresis was carried out and the cells were stained with DAPI solution for 10 min in a dark room. The slides were examined with an Olympus BX63 fluorescence microscope. Tail moment was calculated by using CASP software.

### NADPH/NADP^+^ quantification

Cells were seeded at 6-well plates, allowed to attach, and exposed to 8 Gy irradiation. After 24 h, cells were washed with cold PBS and extracted with NADP^+^/NADPH extraction buffer, followed by centrifugation at 10,000 × g for 10min to remove insoluble material. Samples were deproteinized by filtering through a 10-kDa cut-off spin filter. To detect NADPH, NADP^+^ was decomposed by centrifuging tubes and heating to 60°C for 30 min in a water bath followed by cooling on ice. Samples were quickly spun to remove any precipitates, leaving only NADPH. NADP^+^ and NADPH samples were incubated with a Master Reaction mixture for an appropriate time before the absorbance was measured at 450 nm according to the manufacturer’s protocol (MAK038, Sigma-Aldrich).

### Statistical analysis

Statistical analysis was performed using SPSS 20.0 software, GraphPad Prizm 8, ImageJ. Two-tailed and unpaired Student’s *t*-tests and two-way ANOVA tests were performed to compare differences. The differences in NUPR1 expression levels between the paired HCC tissues and adjacent nontumorous liver tissues in the TCGA database were compared by paired *t*-tests. Pearson correlation analysis was performed to analyze the correlation between two molecules. Survival curves were estimated using the Kaplan-Meier method and compared using the log-rank test. Data were presented as the means ± standard deviation (SD). Statistical significance was defined as a *P*-value less than 0.05. *P < 0.05; **P < 0.01; ***P < 0.001; ns, no significance.

## Results

### NUPR1 acts as a radioresistant oncogene in HCC in vitro

**Fig. 1:**
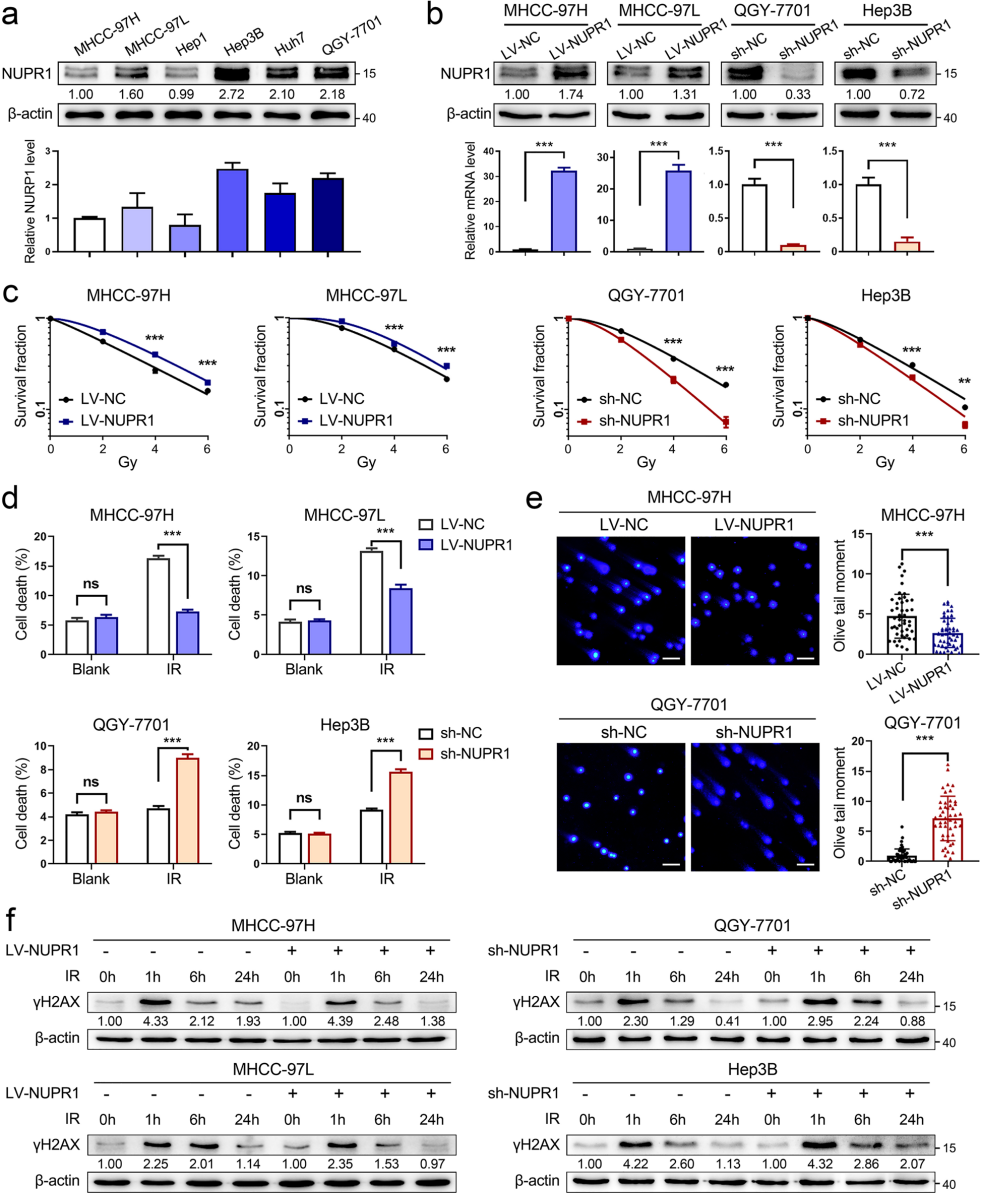
NUPR1 acts as a radioresistant oncogene in HCC in vitro*. ***a** Western blot was used to determine the protein expression of NUPR1 in a panel of HCC cell lines (MHCC-97H, MHCC-97L, Hep1, Hep3B, Huh7, and QGY-7701). Bar graphs presented the quantification of NUPR1 levels in HCC cells (below). **b** The protein and mRNA levels of NUPR1 in MHCC-97H/MHCC-97L cells with NUPR1 overexpression and QGY-7701/Hep3B with NUPR1 knockdown were verified by western blot and qRT-PCR. **c** Colony formation assays were employed in MHCC-97H/MHCC-97L cells with NUPR1 overexpression and QGY-7701/Hep3B cells with NUPR1 knockdown after an increased dose of IR treatment (0, 2, 4, 6 Gy). Survival curves were represented. **d** Bar graphs show the quantification of cell death in NUPR1 overexpression or knockdown cells by staining with 7-AAD after IR treatment (8 Gy). **e** DNA double-strand breaks of NUPR1-overexpressing MHCC-97H and NUPR1-knockdown QGY-7701 cells were detected by comet assays at 24 h after exposure to IR (8 Gy) (left, representative images, scale bar: 50 μm; right, bar graphs indicating the average tail moment per cell). **f** Western blot analysis was used to determine the protein levels of γH2AX in cells with a different NUPR1 expression status at the indicated time points after IR (8 Gy). Data are the mean of biological triplicates and are shown as the mean ± SD. *P* values: *P < 0.05; **P < 0.01; ***P < 0.001 and ns, not significant by two-tailed Student’s *t-*test

To clarify the radioresistant role of NUPR1 on HCC cells, we firstly examined NUPR1 expression in a series of HCC cell lines by western blot and qRT-PCR analyses (Fig. 1a and Additional file 1: Fig.S1a). A previous study demonstrated that γ-irradiation could increase NUPR1 expression and influence DNA damage responses [23]. We evaluated the transcriptional level of NUPR1 in MHCC-97H cells treated with different doses of IR and collected at different timing. However, the level of NUPR1 was not altered after treating X-ray radiation (Additional file 1: Fig. S1b). Next, we established NUPR1-overexpressing cell lines in MHCC-97H, MHCC-97L (LV-NC/LV-NUPR1), and NUPR1-knockdown cell lines in QGY-7701, Hep3B cells (sh-NC/sh-NUPR1) with lentiviral transfection. The protein and mRNA levels of NUPR1 in NUPR1-overexpressing or knockdown HCC cells were verified by western blot and qRT-PCR analyses (Fig. 1b). CCK8 assays indicated that ectopic expression of NUPR1 increased the proliferation of HCC cells. In contrast, knockdown of NUPR1 led to a significant reduction in proliferation (Additional file 1: Fig. S1c). Colony formation assays demonstrated that the clonogenicity of NUPR1-overexpressing cell lines was significantly elevated, whereas NUPR1 knockdown diminished the clonogenicity after IR (Fig. 1c and Additional file 1: Fig. S1d). Moreover, the cell death rates in different NUPR1 expression cell lines were quantified after IR. NUPR1 overexpression significantly repressed cell death, while NUPR1 knockdown increased cell death in response to IR (Fig. 1d).

Since IR can trigger DNA double-strand breaks (DSBs) directly by ionization or indirectly by ROS generation [7]. We treated HCC cells with IR and then measured DSBs by comet assays. As shown in Fig. 1e and Additional file 1: Fig. S1e, compared to control cell lines, LV-NUPR1 cells had shorter tails length, whereas sh-NUPR1 cells extended tails length after IR exposure. Cellular DNA damage was also monitored by γH2AX, a well-known marker of DSBs. We found that NUPR1 overexpression led to a decreased γH2AX expression and a better recovery back to the basal level. In contrast, knockdown of NUPR1 showed the opposite changes in HCC cells after IR treatment (Fig. 1f).

### NUPR1 enhances the radiation resistance of HCC cells in vivo

**Fig. 2:**
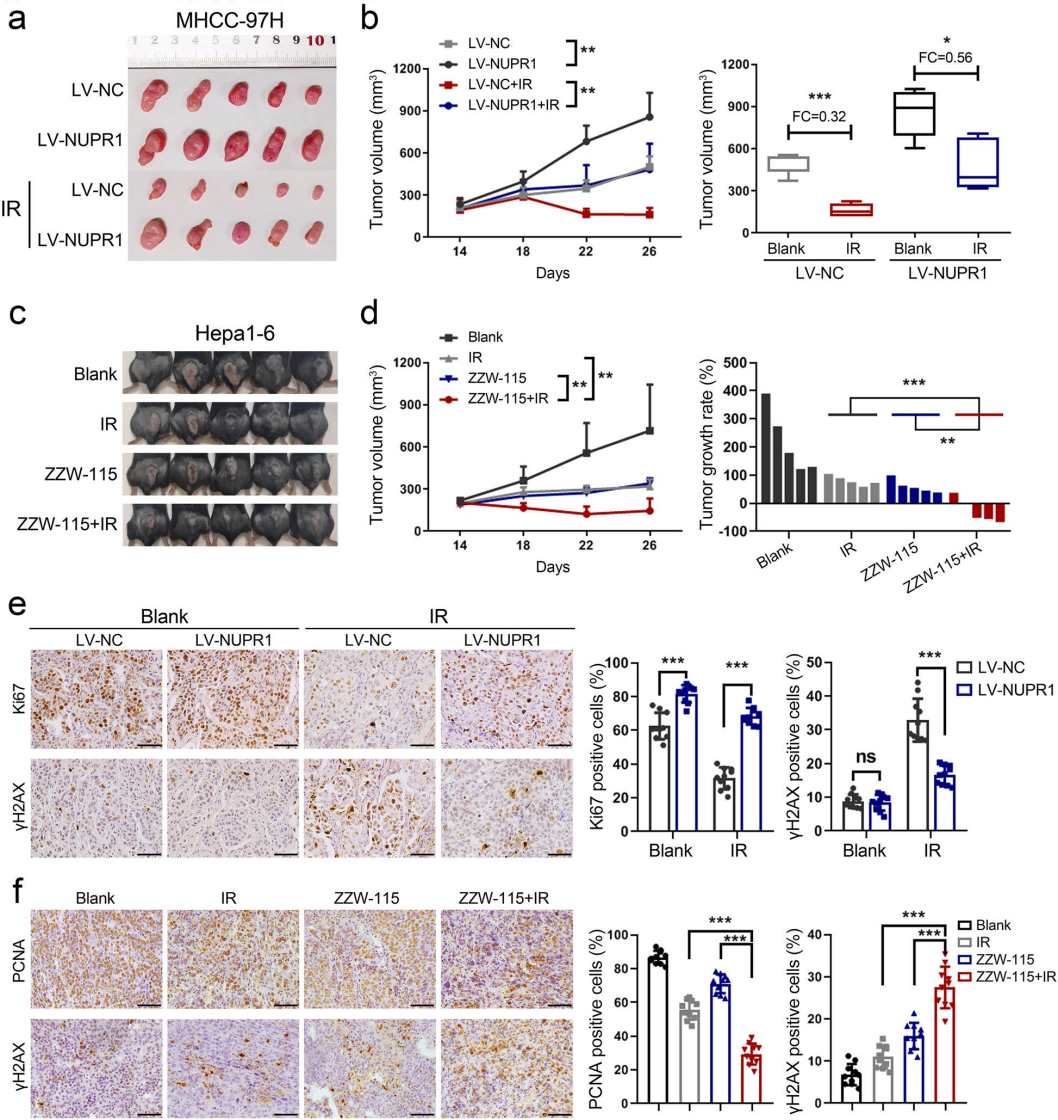
NUPR1 enhances the radiation resistance of HCC cells in vivo*. ***a**, **b** Subcutaneous tumor formation in nude mice was established with NUPR1-overexpressing or control MHCC-97H cells (*n* = 5/group). Tumor sizes were measured every 4 days using calipers (left, representative tumor samples; middle, growth curves of subcutaneous tumors; right, the statistical graph of tumor volumes). **c** Representative images of each group were photographed at the end of the experiment. **d** Growth curves of subcutaneous tumors (left) and tumor growth rates of mice treated with IR, ZZW-115, or IR plus ZZW-115 were represented (right). **e** IHC images of Ki67 and γH2AX expression in xenograft tumors derived from MHCC-97H cells with NUPR1 overexpression or empty vector were represented (left, scale bar: 50 μm). The positive stain (in percentages) was analyzed (right). **f** IHC images of PCNA and γH2AX expression in xenograft tumors derived from Hepa1-6 cells with different treatments were shown (left, scale bar: 50 μm). The positive stain (in percentages) was analyzed (right). Data are the mean of biological triplicates and are shown as the mean ± SD. *P* values: *P < 0.05; **P < 0.01; ***P < 0.001 by two-tailed Student’s *t*-test, or by two-way ANOVA

To extend the in vitro results, we explored the radioresistant effect of NUPR1 by using a xenograft model. As shown in Fig. 2a, b, NUPR1-overexpressing MHCC-97H cells enhanced tumor growth in nude mice. After being treated with IR, the NUPR1-overexpressing tumors exhibited minor regression and larger volumes than control tumors. NUPR1 inhibitor ZZW-115 was proved to sensitize pancreatic cancer cells to genotoxic agents, including γ-irradiation [24]. Therefore, combined treatment with ZZW-115 and IR was utilized in C57/BL6 mice after implanting murine Hepa1-6 HCC cells. We observed a synergistic tumor regression in xenografts following the combined treatment of ZZW-115 and IR (Fig. 2c, d). IHC staining showed that Ki67 was relatively unregulated in the NUPR1-overexpressing tumors compared with control tumors after IR exposure, whereas γH2AX was significantly downregulated (Fig. 2e). In addition, another marker of proliferation, PCNA was repressed considerably in tumors with combined ZZW-115 and IR treatment, while γH2AX showed opposite changes (Fig. 2f). Collectively, all of these results indicated that NUPR1 played a crucial role in the radiation resistance of HCC.

### NUPR1 inhibits ROS generation and oxidative stress via CYPs in HCC cells

**Fig. 3:**
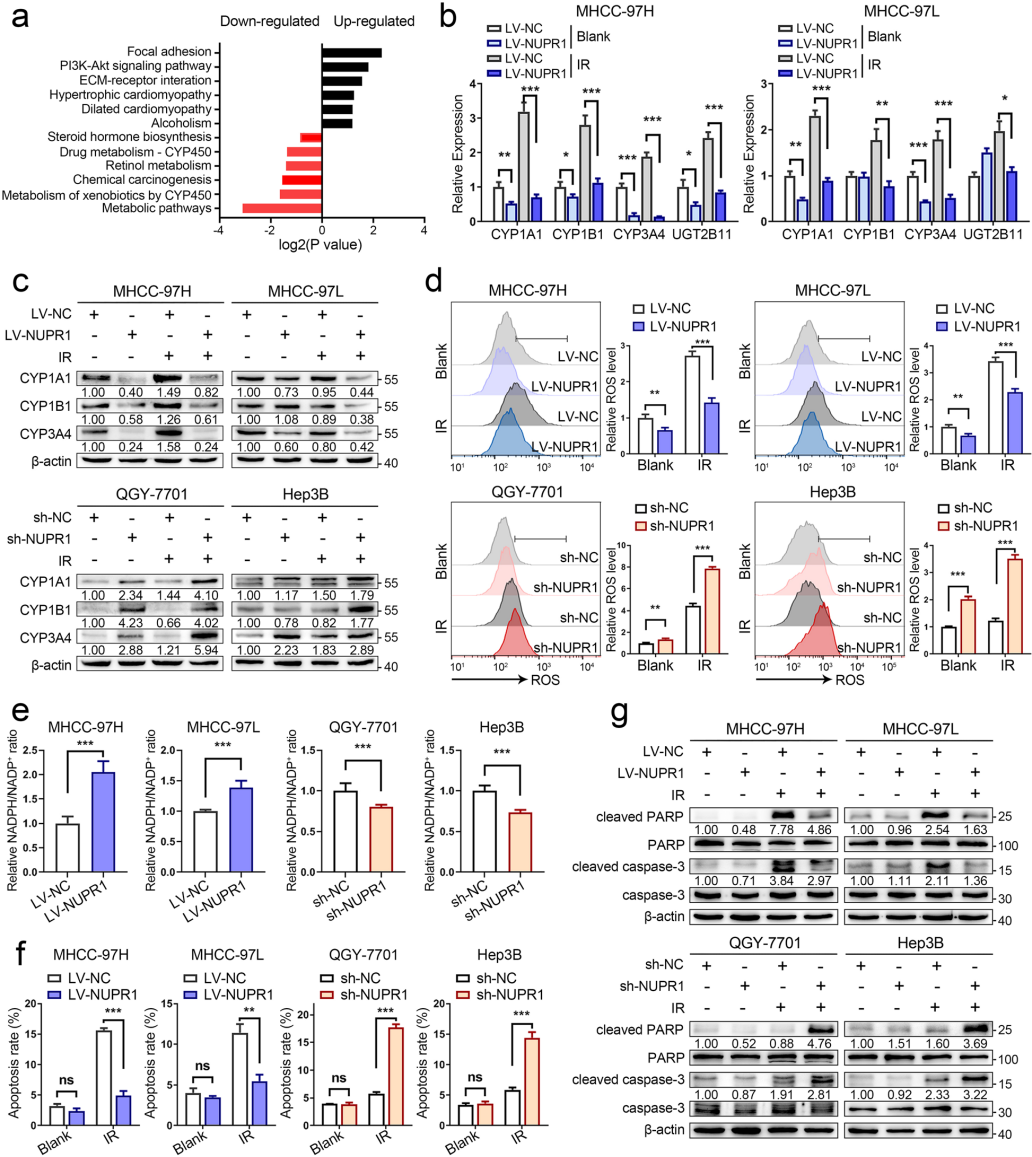
NUPR1 inhibits ROS generation and oxidative stress via CYPs in HCC cells. **a** KEGG enrichment analysis of differentially expressed genes between NUPR1-overexpressing and control MHCC-97H cells showed that the metabolic pathway mediated by cytochrome P450 was downregulated in LV-NUPR1 cells. **b** The mRNA levels of genes included in CYP superfamily in MHCC-97H/MHCC-97L cells transfected with LV-NUPR1 or LV-NC were analyzed by qRT-PCR. Individual RNA values were normalized to β-actin values. **c** Western blot analysis was used to detect the protein expression of CYP1A1, CYP1B1, and CYP3A4 in NUPR1 overexpressing or knockdown cell lines treated with or without IR (8 Gy). **d, e** ROS levels and NADPH/NADP^+^ ratio in stable NUPR1 overexpressing or knockdown cell lines were measured after exposure to 8 Gy of IR. **f** Bar graphs show the relative levels of apoptotic cells after IR treatment (8 Gy) by staining with annexin V and DAPI in indicated cells. **g** Western blot was used to detect the protein levels of total/cleaved caspase-3 and total/cleaved PARP in different NUPR1 expressing cell lines treated with or without IR (8 Gy). Data are the mean of biological triplicates and are shown as the mean ± SD. *P* values: *P < 0.05; **P < 0.01; ***P < 0.001 and ns, not significant by Student’s *t-*test

In order to uncover the radioresistant mechanism of NUPR1 on HCC, RNA sequencing was performed and screened several upregulated (*n* = 263, FC > 1.5, *P* < 0.05) and downregulated (*n* = 269, FC < 0.67, *P* < 0.05) genes in LV-NUPR1 MHCC-97H cells compared with LV-NC cells. We utilized KEGG pathway enrichment analysis and identified that the cytochrome P450 (CYP)-mediated metabolism pathway was notably downregulated in LV-NUPR1 cells (Fig. 3a). Western blot and qRT-PCR analyses showed that the expressions of several cytochrome P450 enzymes (CYPs) such as CYP1A1, CYP1B1, and CYP3A4 were significantly reduced in cells with NUPR1 overexpression (Fig. 3b, c). In contrast, knockdown of NUPR1 resulted in a notable increase in their expressions (Fig. 3c and Additional file 1: Fig. S2a).

Since CYP enzymes are responsible for detoxifying toxic metabolites and lead to the formation of ROS, such as superoxide anion (O_2_^−^) and hydrogen peroxide (H_2_O_2_) [25]. Therefore, we focused on the potential regulation of NUPR1 in ROS generation. Our results showed that NUPR1 overexpression prevented the formation of ROS compared with control cells, whereas NUPR1 knockdown induced higher ROS levels after IR (Fig. 3d). NADPH, a scavenger of ROS, was reported to be consumed by CYP [25]. We detected the ratio of NADPH/NADP^+^ in cells with a different NUPR1 expression status. The results showed that LV-NUPR1 cells exhibited a remarkable increase in NADPH/NADP^+^ ratio, while an opposite change was seen in sh-NUPR1 cells upon IR (Fig. 3e). High amount of ROS production can result in a series of oxidative stress such as apoptosis, lipid peroxidation, and DNA oxidative damage [8]. As expected, ectopic NUPR1 expression in MHCC-97H and MHCC-97L cells significantly inhibited cell apoptosis and lipid peroxidation, whereas silencing of NUPR1 exhibited opposite results after IR treatment (Fig. 3f and Additional file 1: Fig. S2b-d). Molecular markers of apoptosis, such as cleaved PARP and cleaved caspase-3, were decreased by NUPR1 overexpression but elevated in NUPR1-knockdown cells in response to IR (Fig. 3g). Altogether, these results demonstrated that NUPR1 could attenuate CYPs-mediated ROS generation and the downregulation of ROS may enhance the radioresistance of HCC.

### ROS generated by CYPs is indispensable for IR-induced cytotoxicity in cells with NUPR1 inactivation

**Fig. 4:**
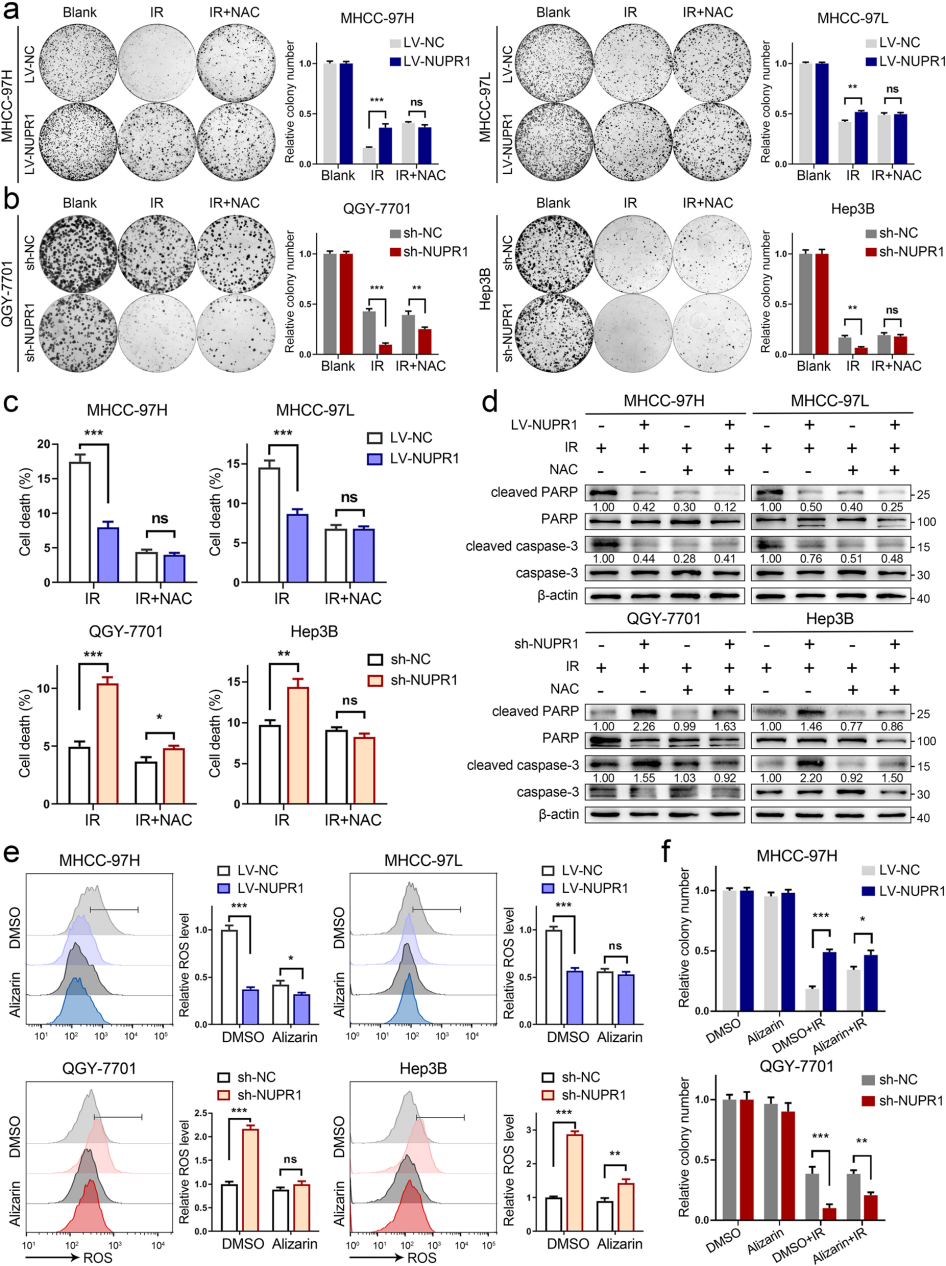
ROS generated by CYPs is indispensable for IR-induced cytotoxicity in cells with NUPR1 inactivation. **a**, **b** Representative images of colony formation were displayed in NUPR1-overexpressing MHCC-97H/MHCC-97L (3000 cells) and NUPR1-knockdown QGY-7701/Hep3B (500 cells and 3000 cells, respectively) pretreated with 5 mM NAC followed by exposure to 6 Gy of IR (left). The survival data were normalized to those of unirradiated control cells (right). **c** Quantification of cell death was employed in LV-NC/LV-NUPR1 or sh-NC/sh-NUPR1 cell lines pretreated with or without 5 mM NAC followed by exposure to 8 Gy of IR. **d** Western blot analysis was utilized to determine the expression levels of total/cleaved caspase-3 and total/cleaved PARP in the indicated cells pretreated with or without NAC upon IR (8 Gy). **e** Relative ROS levels were measured in NUPR1 overexpressing or knockdown cell lines pretreated with or without 20 μM alizarin followed by 8 Gy of IR. **f** Colony formation assays were applied in stably transfected NUPR1 overexpression or knockdown cells after IR (6 Gy) or combination with 20 μM alizarin treatment. Data are the mean of biological triplicates and are shown as the mean ± SD. *P* values: *P < 0.05; **P < 0.01; ***P < 0.001 and ns, not significant by Student’s *t-*test

Next, we sought to validate the potential effect of ROS in NUPR1-mediated radioresistance in HCC. We utilized ROS scavenger, N-acetyl-L-cysteine (NAC), to examine its impact on the colony formation of cells with a different NUPR1 expression status under IR. As shown in Fig. 4a, b, relative to LV-NUPR1 cells, treatment with NAC significantly restored clonogenicity in LV-NC cells, while a reduced clonogenicity in NUPR1-knockdown cells was also abrogated by adding NAC. The cell lines with NUPR1 silencing were more vulnerable to cell death upon exposure to IR. When adding NAC, the cell death rates of these cell lines had a significant restoration (Fig. 4c). Furthermore, IR-induced oxidative stress, including apoptosis and lipid peroxidation, also had a remarkable restoration following a combined treatment of IR and NAC in LV-NC cells and sh-NUPR1 cells (Additional file 1: Fig. S3a-c). Consistent with flow cytometry data, the expression of cleaved PARP and cleaved caspase-3 in indicated cells were alleviated by NAC treatment following IR (Fig. 4d).

A previous study demonstrated that alizarin strongly inhibited the activities of CYP1A1 and CYP1B1 [26]. To specifically evaluate the functional role of ROS derived from CYPs’ catalysis. We applied a concentration gradient of alizarin to culture MHCC-97H and MHCC-97L cells followed by IR exposure. As shown in Additional file 1: Fig. S4a, alizarin significantly antagonized ROS generation in tumor cells in a dose-dependent manner. Additionally, relative to LV-NUPR1 and sh-NC cells, alizarin significantly reduced the ROS levels in LV-NC and sh-NUPR1 cells (Fig. 4e). In line with ROS analyses, increased ROS-mediated apoptosis in cell lines with NUPR1 inactivation was abrogated by alizarin upon IR treatment (Additional file 1: Fig. S4b, c). Colony formation assays revealed that alizarin could modestly increase the clonogenicity of LV-NC MHCC-97H and sh-NUPR1 QGY-7701 cells in response to IR (Fig. 4f and Additional file 1: Fig. S4d). The results supported the notion that excessive ROS generated by CYPs contributed to oxidative stress and radiation sensitivity in tumor cells with NUPR1 inactivation. NAC and alizarin enabled tumor cells to survive upon IR treatment by eliminating ROS.

### NUPR1 binds to AhR and promotes degradation of AhR in the autophagy-lysosome pathway

**Fig. 5:**
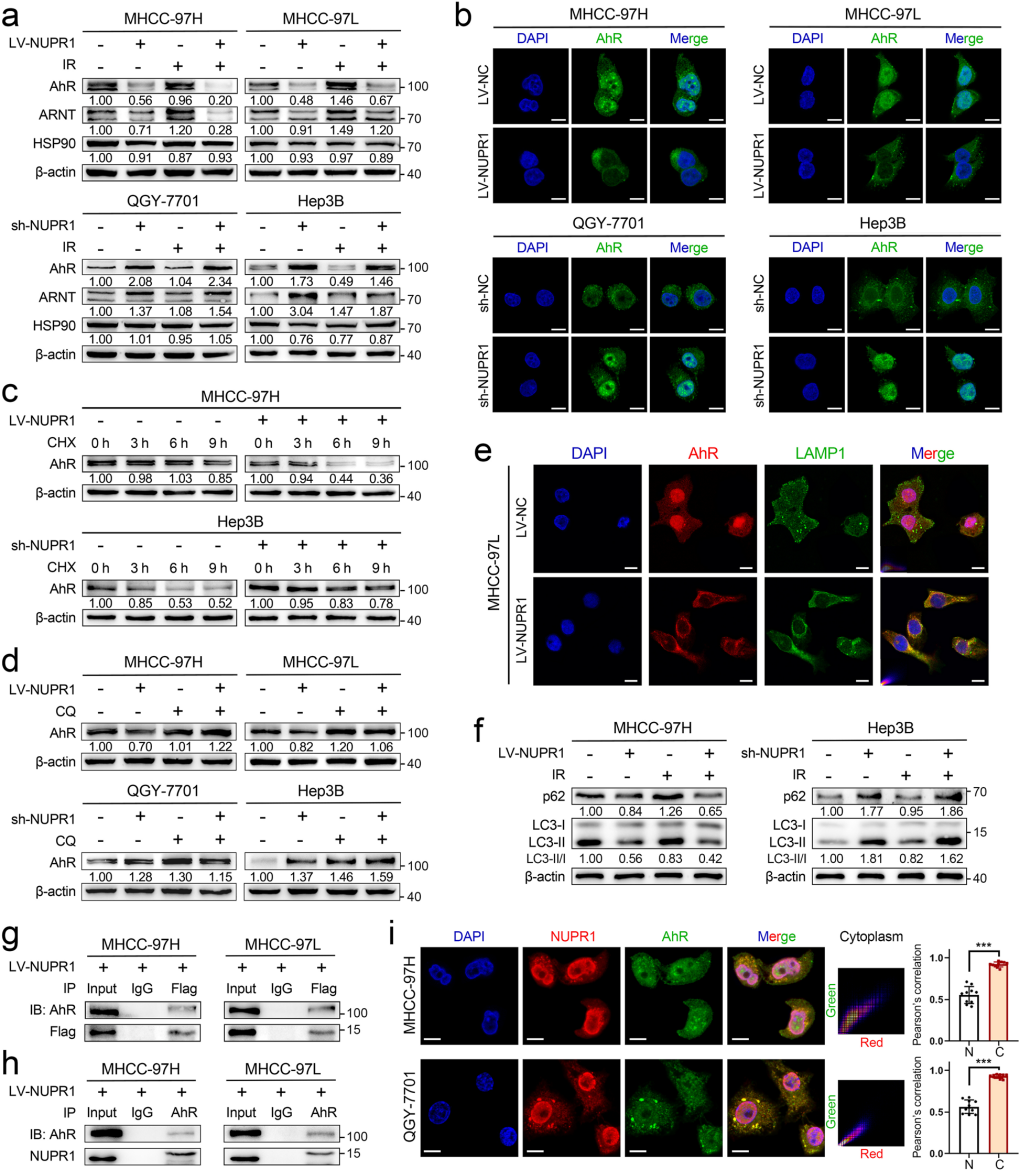
NUPR1 binds to AhR and promotes degradation of AhR in the autophagy-lysosome pathway. **a** Western blot was used to examine the protein levels of AhR, ARNT, and HSP90 in NUPR1-overexpressing MHCC-97H/MHCC-97L cells and NUPR1-knockdown QGY-7701/Hep3B cells with or without IR (8 Gy). **b** Immunofluorescence staining was performed to determine the location of AhR (green) in the indicated cell lines. The nuclei were counterstained with DAPI (blue). Scale bar: 10 μm. **c** Western blot was used to detect AhR levels in indicated cell lysates collected at different timing (0, 3, 6, 9 h) after 20 μg/mL CHX treatment. **d** Western blot of AhR protein was performed in cell lysates from different NUPR1 expression status cell lines with or without 20 μM CQ treatment. **e** Representative immunofluorescence images show the distribution of AhR (red), LAMP1 (green), and DAPI (blue) in LV-NC and LV-NUPR1 MHCC-97L cells. Scale bar: 10 μm. **f** The levels of LC3-II and p62 in cells with a different NUPR1 expression were determined by western blot. **g** The anti-Flag and anti-IgG products after incubating with lysates from MHCC-97H/MHCC-97L ectopically expressed Flag-tagged NUPR1 were used to detect Flag-NUPR1 and AhR protein by western blot. **h** Western blot analysis of NUPR1 and AhR in anti-AhR and anti-IgG products was performed. Cell lysates were immunoprecipitated with antibodies against AhR or IgG. **i** Representative immunofluorescence images of QGY-7701/MHCC-97H cells show the staining of NUPR1 (red), AhR (green), and nuclei counterstained with DAPI (blue) (left). Scale bar: 10 μm. Pearson correlation of the signal intensity of indicated molecules in different subcellular locations was quantified by ImageJ software (N/nucleus, C/cytoplasm, right)

AhR/ARNT complex is well known for transcriptional regulation of CYPs, such as CYP1A1 and CYP1B1 [27]. Upon activation by various cellular stress, the aryl hydrocarbon receptor (AhR) dissociates from HSP90 and heterodimerizes with the aryl hydrocarbon receptor nuclear translocator (ARNT) in the nucleus to regulate the transcriptional expression of target genes [28]. Hence, we hypothesized that NUPR1 might regulate AhR/ARNT complex to mediate CYPs expression and subsequent ROS formation upon IR. To test this hypothesis, we examined the protein expression of the primary molecules in the AhR/CYPs pathway and found that AhR and ARNT were significantly reduced in NUPR1-overexpressing cells, whereas they increased in cells with NUPR1 knockdown (Fig. 5a). Interestingly, the mRNA levels of AhR and ARNT did not display consistent changes (Additional file 1: Fig. S5a). Moreover, NUPR1 overexpression decreased the nuclear expression of AhR and ARNT, while NUPR1 knockdown caused an opposite change (Additional file 1: Fig. S5b). Immunofluorescent (IF) staining of LV-NC cells showed that AhR was mainly located in the nucleus. Ectopic NUPR1 expression increased the cytoplasmic distribution of AhR and weakened the colocalization between AhR and DAPI. Conversely, NUPR1 knockdown increased the nuclear translocation of AhR and overlapped with DAPI (Fig. 5b). However, the distribution of ARNT was primarily located in the nucleus and was not affected by different NUPR1 expressions (Additional file 1: Fig. S5c).

In light of the reverse regulation of AhR by NUPR1, we firstly treated cells with cycloheximide (CHX) to determine the stability of AhR. Our results showed that the half-life periods of AhR were much shorter in cells with NUPR1 overexpression than were in control cells, whereas AhR protein degraded slower in NUPR1 knockdown cells (Fig. 5c and Additional file 1: Fig. S5d). As we know, lysosome acts as a recycling center for regulating the degradative endpoint of the endosomal pathway. Therefore, we treated cells with selective lysosomal inhibitor chloroquine (CQ) or proteasome inhibitor MG132. Results revealed that CQ treatment could significantly increase AhR levels in LV-NUPR1 and sh-NC cells, while a slight upregulation of AhR was observed in LV-NC and sh-NUPR1 cells (Fig. 5d). Treatment with CQ also restored the nuclear expression of AhR in indicated cells (Additional file 1: Fig. S6a). Nevertheless, MG132 treatment did not alter the AhR protein levels (Additional file 1: Fig. S6b). Furthermore, overexpression of NUPR1 in tumor cells significantly increased the distribution of AhR to lysosome marker LAMP1 (Fig. 5e and Additional file 1: Fig. S6c). Previous works revealed that NUPR1 regulated autophagic flux and autolysosomal efflux in multiple cancer cells [17, 18, 29]. Autophagy is a conserved self-eating process that cells perform to allow degradation by formatting a double-membrane containing the sequestered cytoplasmic material and ultimately fuses with lysosome [30]. To verify the involvement of autophagy in AhR’s degradation, we analyzed the expression of two autophagy-related proteins, LC3-II and p62. Results showed that NUPR1 knockdown caused a significant increase of LC3-II and p62 expression levels, suggesting the autophagic flux was impeded, whereas the opposite change was seen in NUPR1-overexpressing cells (Fig. 5f and Additional file 1: Fig. S6d). Treatment with autophagy inhibitors bafilomycin A1 (BafA1) can also restore AhR expression in LV-NUPR1 MHCC-97H and sh-NC Hep3B cells (Additional file 1: Fig. S6e). These results suggested that NUPR1 modulated the protein stability of AhR via the autophagy-lysosome pathway.

To gain insight into the potential interaction between NUPR1 and AhR, we performed co-immunoprecipitation (Co-IP) combined with liquid chromatography-tandem mass spectrometry (LC-MS/MS) using Flag-tagged NUPR1-overexpressing cells lysates and anti-Flag antibody. AhR was identified as one of the NUPR1-interacting proteins (Additional file 1: Fig. S6f). Co-IP and western blot were applied to confirm the interaction between NUPR1 and AhR (Fig. 5g). Reciprocal Co-IP assays were conducted with antibodies against AhR to co-precipitate NUPR1 and further confirmed the interaction of these two proteins (Fig. 5h). Using an anti-AhR antibody to incubate the lysate from QGY-7701 and Hep3B cells, the consistent results validated the endogenous interaction between NUPR1 and AhR in HCC cells (Additional file 1: Fig. S6g). Additionally, we analyzed the subcellular location of NUPR1 and AhR by IF staining. NUPR1 signal primarily overlapped with AhR in the cytoplasm but not in the nucleus (Fig. 5i). These results demonstrated that NUPR1 bound to AhR and modulated its cellular distribution, which may downregulate IR-induced oxidative stress by inhibiting AhR/CYP signaling axis.

### AhR/CYP signal axis is required for NUPR1-mediated radioresistance in HCC

**Fig. 6:**
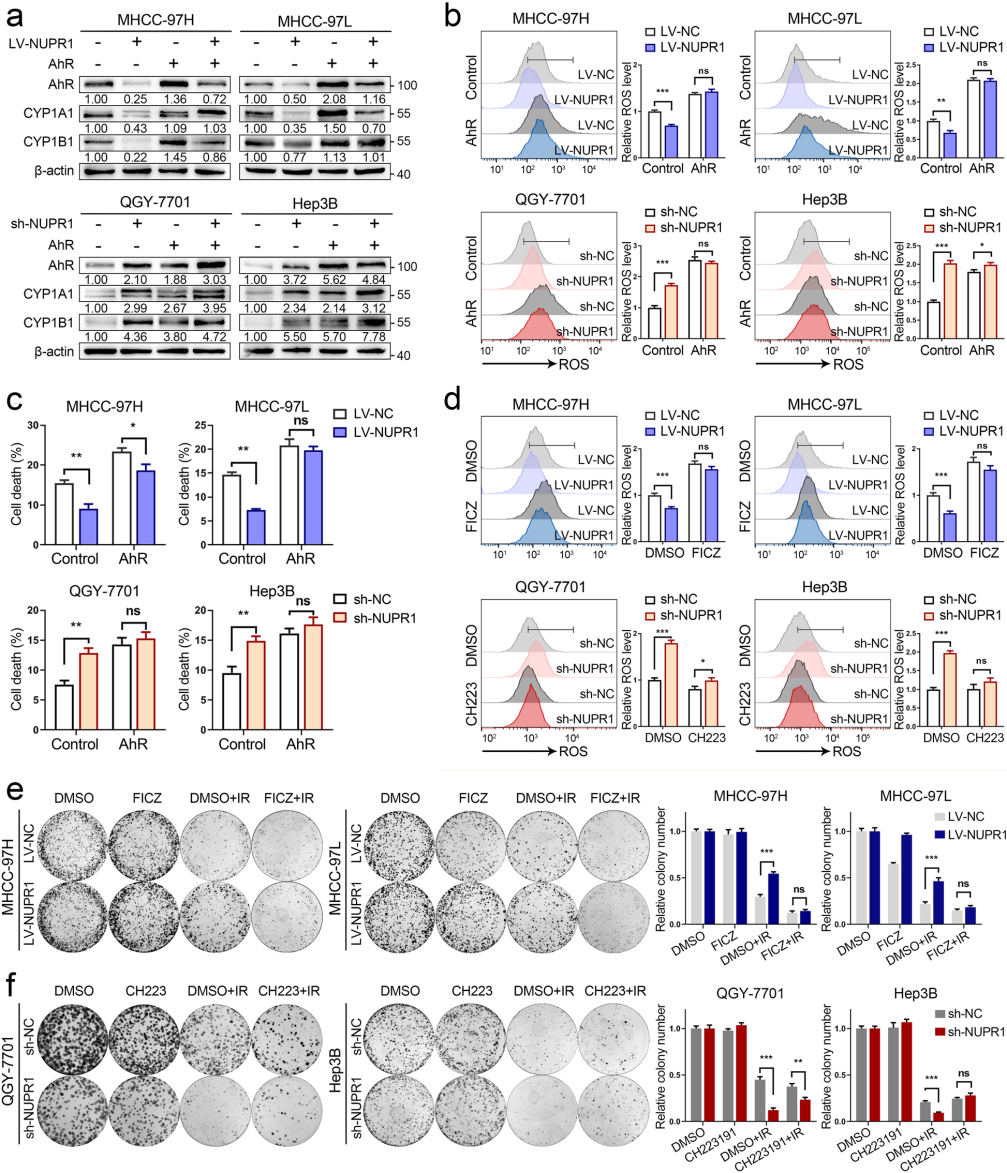
AhR/CYP signal axis is required for NUPR1-mediated radioresistance in HCC. **a** Western blot analysis was utilized to examine the expression levels of AhR, CYP1A1, and CYP1B1 in different NUPR1 expressing cell lines transfected with AhR-overexpressing vector or control vector. **b** Relative ROS levels in different NUPR1 expressing cells with AhR overexpression or negative control were measured after exposure to 8 Gy of IR. **c** Quantification of cell death in indicated cells with AhR overexpression or negative control was measured after IR (8 Gy). **d** Cells with different NUPR1 expressions were pretreated with 5 μM FICZ or CH223191 for 24 h, followed by being exposed to 8 Gy of IR. The cells were collected to measure relative ROS levels. **e, f** Colony formation assays were performed in stably transfected NUPR1 overexpression or knockdown cell lines pretreated with FICZ or CH223191, followed by exposure to 6 Gy of IR. Data are the mean of biological triplicates and are shown as the mean ± SD. *P* values: *P < 0.05; **P < 0.01; ***P < 0.001 and ns, not significant by two-tailed Student’s *t-*test

To further explore the impact of AhR on the NUPR1-mediated radioresistance of HCC cells. We generated AhR-overexpressed plasmids and transfected them into cells with a different NUPR1 expression status, followed by exposure to IR. In our study, AhR overexpression led to an upregulation of CYP1A1 and CYP1B1 in HCC cells. In contrast, a larger increase of CYPs expression was seen in LV-NUPR1 and sh-NC cells (Fig. 6a). Next, our results showed that ectopic expression of AhR significantly improved ROS generation and cell death rates in LV-NUPR1 cells and sh-NC cells, whereas a slight upregulation of ROS and cell death was seen in LV-NC cells and sh-NUPR1 cells upon IR (Fig. 6b, c). Cleaved PARP and cleaved caspase-3 were increased in LV-NUPR1 cells and sh-NC cells by AhR-overexpressing after IR treatment (Additional file 1: Fig. S7a).

Since the pharmacological intervention of AhR is available, we supposed that AhR might serve as a potential target for reversing the radioresistant role mediated by NUPP1 in HCC. Our results showed that pharmacological activation with AhR agonist FICZ significantly elevated ROS levels in cells with NUPR1-overexpression, while specific inhibiting AhR by CH223191 resulted in a suppression of ROS levels in NUPR1-knockdown cells in response to IR (Fig. 6d). The plate colony formation assays revealed that FICZ treatment significantly inhibited the clonogenic survival in LV-NUPR1 cells, whereas CH223191 treatment could restore the clonogenicity in sh-NUPR1 cells after IR (Fig. 6e, f). IHC staining of xenograft tumors derived from MHCC-97H cells showed that the expression levels of AhR and CYP1A1 were lower in LV-NUPR1 tumors than in LV-NC tumors. Upon IR treatment, the expression of malondialdehyde (MDA), a lipid peroxidation product, was significantly increased in LV-NC tumors, while a slight upregulation was seen in LV-NUPR1 tumors (Additional file 1: Fig. S7b). These results implicated that NUPR1 may diminish ROS generation and oxidative stress via AhR/CYP signal axis in HCC cells under IR treatment.

### NUPR1 is upregulated in HCC tissues and predicts radiotherapeutic resistance of HCC

**Fig. 7:**
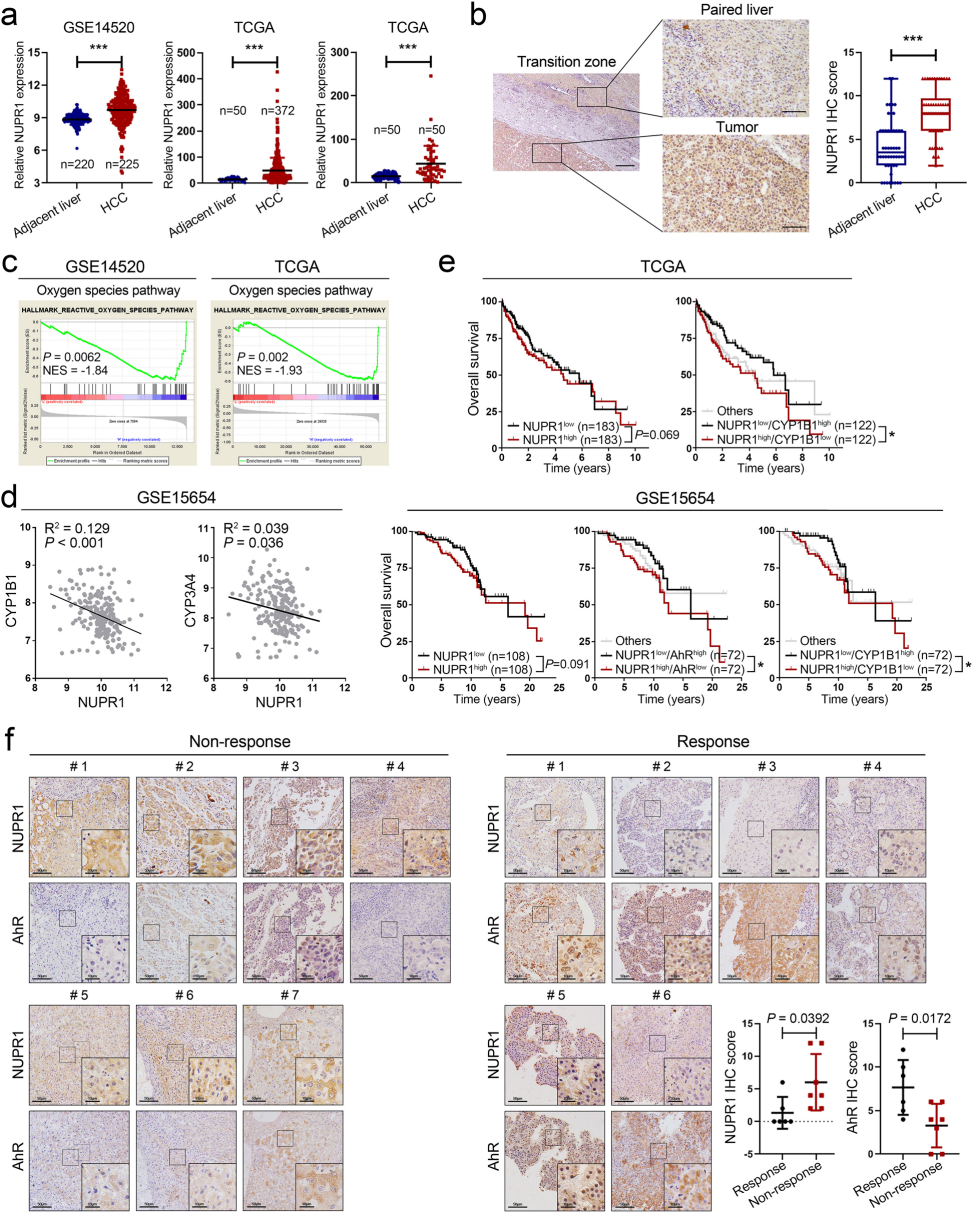
NUPR1 is upregulated in HCC tissues and predicts radiotherapeutic resistance of HCC. **a** NUPR1 mRNA expression in unpaired HCC tissues and non-tumor liver tissues from TCGA and GSE14520 datasets were shown (left and middle). NUPR1 mRNA expression in 50 paired HCC tissues and the adjacent matched noncancerous tissues was displayed on the right. **b** Representative IHC images show the NUPR1 expression in HCC (*n* = 50) and corresponding adjacent liver tissues (*n* = 50, left). Scale bar: 200 μm in 4 × magnification and 50 μm in 20 × magnification. Quantification of IHC score was shown in boxplot (right). **c** Gene set enrichment analysis (GSEA) with relatively low-expressed versus high-expressed NUPR1 from TCGA LIHC and GSE14520 datasets showed that the oxygen species pathway was positively correlated with NUPR1 expression. **d** The mRNA level of NUPR1 was negatively correlated with the CYP1B1 and CYP3A4 expression in the GSE15654 dataset by using Pearson correlation analysis. **e** Kaplan-Meier survival analysis was used to assess the correlation between NUPR1 expression or combined with AhR, CYP1B1 level, and the overall survival of HCC patients in TCGA LIHC dataset (*n* = 366) and GES15654 dataset (*n* = 216). **f** NUPR1 and AhR expression in 13 HCC tissues from the patients who accepted RT treatment were analyzed by IHC. Representative IHC images (left) and quantified IHC score (right) are shown

To further assess the potential correlation of NUPR1 and clinical data of HCC patients, we analyzed NUPR1 mRNA expression in GEO and TCGA databases. As shown in Fig. 7a, NUPR1 mRNA expression was significantly higher in HCC tissues than in benign counterparts. We then verified the protein levels of NUPR1 by IHC and found that NUPR1 was upregulated in HCC tissues relative to the matched adjacent liver tissues (Fig. 7b and Additional file 1: Fig. S8a). Furthermore, we performed gene-set enrichment (GSEA) analysis with RNA sequencing data from TCGA LIHC and GSE14520 datasets. Results showed that the ROS pathway and glutathione metabolism pathway were enriched in the HCC specimens with relatively high expression of NUPR1 (Fig. 7c and Additional file 1: Fig. S8b). Pearson correlation analysis showed that NUPR1 mRNA level was negatively correlated with CYP1B1 and CYP3A4 mRNA levels in HCC tissues from the GSE15654 dataset (Fig. 7d). Kaplan–Meier survival analysis indicated that the patients with high expression of NUPR1 and low-expressed AhR or CYP1B1 showed significantly worse overall survival in TCGA LIHC and GSE15654 datasets. The mRNA expression of NUPR1 was correlated with worse overall survival in HCC patients but did not have a significant statistic value (Fig. 7e).

Given that NUPR1 conferred a radioresistant effect to HCC via the downregulation of AhR, we detected the expression of NUPR1 and AhR in 13 HCC samples from patients who underwent hepatectomy or liver biopsy guided by ultrasound before RT. HCC samples were collected from the Department of Pathology at Nanfang Hospital, and the detailed clinical information was published in our previous research [6]. Among these patients, six were defined as “non-response” who had tumor recurrence or metastasis after RT treatment within half a year, while seven were classified as “response” without tumor progression after RT. Our IHC results showed that the NUPR1 level of HCC was higher in “non-response” patients relative to “response” patients, while the levels of AhR exhibited an opposite change and had a lower significant statistic value (Fig. 7f). Taken altogether, our finding supported a role of the NUPR1/AhR/CYP signal axis in promoting radioresistance of HCC and suggested that NUPR1 and AhR might serve as potential targets for the development of radiation sensitization in HCC.

## Discussion

During RT, ROS is generated from various sources, for example, the radiolysis of water, electron transport chain in mitochondria, and ROS-induced enzymes, including NADPH oxidase, lipoxygenases, and CYP [31, 32]. Excessive ROS levels induce oxidative stress by reacting with lipids, proteins, and DNA to cause lipid peroxidation, protein misfolding, and DNA strand breaks [8]. ROS maintained at a moderate level is crucial for cancer cells to prevent oxidative damage [10]. Studies reported that many complementary approaches that enhanced ROS production were applied to improve radiosensitivity in cancers [33, 34]. Recently, NUPR1 has been demonstrated to impact ROS production and redox homeostasis in multiple types of cancers [19, 20]. In this study, our results revealed that NUPR1 potently reduced ROS generation by attenuating CYP catalytic activity, therefore enhancing cell viability during IR. When treated with NAC, NUPR1-silencing cells significantly repressed ROS levels and oxidative stress upon IR exposure. The results strongly supported the role of NUPR1 in alleviating ROS formation and oxidative stress after IR treatment in HCC.

NUPR1 is widely reported to act as an oncogene in several types of cancers and regulates a series of downstream genes via interacting with transcription factors [15–18]. Consistent with previous findings, we found that NUPR1 was upregulated in HCC tissues compared with adjacent liver tissues, and NUPR1 overexpression significantly enhanced the proliferation of HCC cells. Notably, we found that CYP-mediated metabolism was the downstream signal upon ectopic expression of NUPR1 in HCC. CYP enzymes are not only known for regulating substrate oxidation, particularly in phase I metabolism of xenobiotics, but also involved in the biosynthesis of cholesterol, fatty acids, and steroid hormones [35]. Early works in CYP biology revealed that CYP could produce ROS due to the inefficiency of electron transfer from NADPH to CYP for monooxygenation of substrate, which was known as “reaction uncoupling”. Besides, continued production of ROS is inevitable for NADPH consumption both in presence and in absence of substrates [36, 37]. A previous study illustrated that cytochrome P450 oxidoreductase (POR), an enzyme required for electron transfer from NADPH to CYPs, was indispensable for lipid peroxidation in ferroptotic cell death of cancer cells [38]. Based on previous findings and our results, it was reasonable to propose that the downregulation of CYPs and ROS levels mediated by NUPR1 could be a novel mechanism for the radioresistance of HCC. Simultaneously, we observed that CYPs expression was elevated by IR treatment in HCC cells, which was similar to the results found in the previous studies [39]. But how IR regulates the activation of CYPs is unclear and warrants further investigations.

Mechanistically, we found that NUPR1 bound to and regulated the degradation process of AhR. As a ligand-activated transcription factor, AhR enables cells to adapt to changing environments and exerts a critical role in the development of cancer [40, 41]. Upon ligand binding, AhR translocates into the nucleus and forms with ARNT as a heterodimer to induce the transcription of target genes [27]. Next to xenobiotics, natural ligands derived from endogenous metabolisms, such as tryptophan catabolite 6-formylindolo[3,2-b]carbazole (FICZ) and kynurenine (Kyn), are potent AhR agonist [42, 43]. Initially, several studies proved that AhR mediated the toxic effect of organic pollutants via the transcriptional induction of CYP and sustained generation of ROS [44]. In this study, ectopic expression of NUPR1 in HCC cells resulted in a downregulation of AhR and impaired its nuclear translocation. Genetic upregulation and pharmacological activation of AhR significantly improved intracellular ROS levels and radiosensitivity in NUPR1-overexpressing cell lines. Treatment with AhR inhibitor CH223191 strikingly restored the radioresistant effect in HCC cells. These results implicated that AhR was indispensable for NUPR1 restraining ROS generation and oxidative stress during IR.

Accumulating evidence highlights the role of AhR in cancer development encompasses both pro- and anti-tumorigenic activities. AhR was proposed to display tumor suppressor function in multiple cancers associated with the brain, liver, digestive system, and skin (melanoma) [45]. Targeting AhR must be dependent on tumor-specific AhR expression. Our study revealed that AhR was relatively low expressed in radiotherapy non-response HCC patients, which may be indicated to enhance the radiosensitivity of HCC by pharmacological activation of AhR. A study demonstrated that knockdown of p23 could drive the autophagy-mediated degradation of AhR [46], although it was well known that AhR was degraded by ubiquitin-proteasome after translocating into nucleus [47]. Moreover, NUPR1 was proven as a potent regulator of autolysosome processing in the late stages. Our data suggested that NUPR1 caused the induction of autophagy flux and enhanced the protein instability of AhR via the autophagy-lysosome pathway, but ANRT might not be directly regulated by NUPR1. The detailed biological mechanism of the interaction between NUPR1 and AhR still needs more effort to dissect.

## Conclusions

**Fig. 8:**
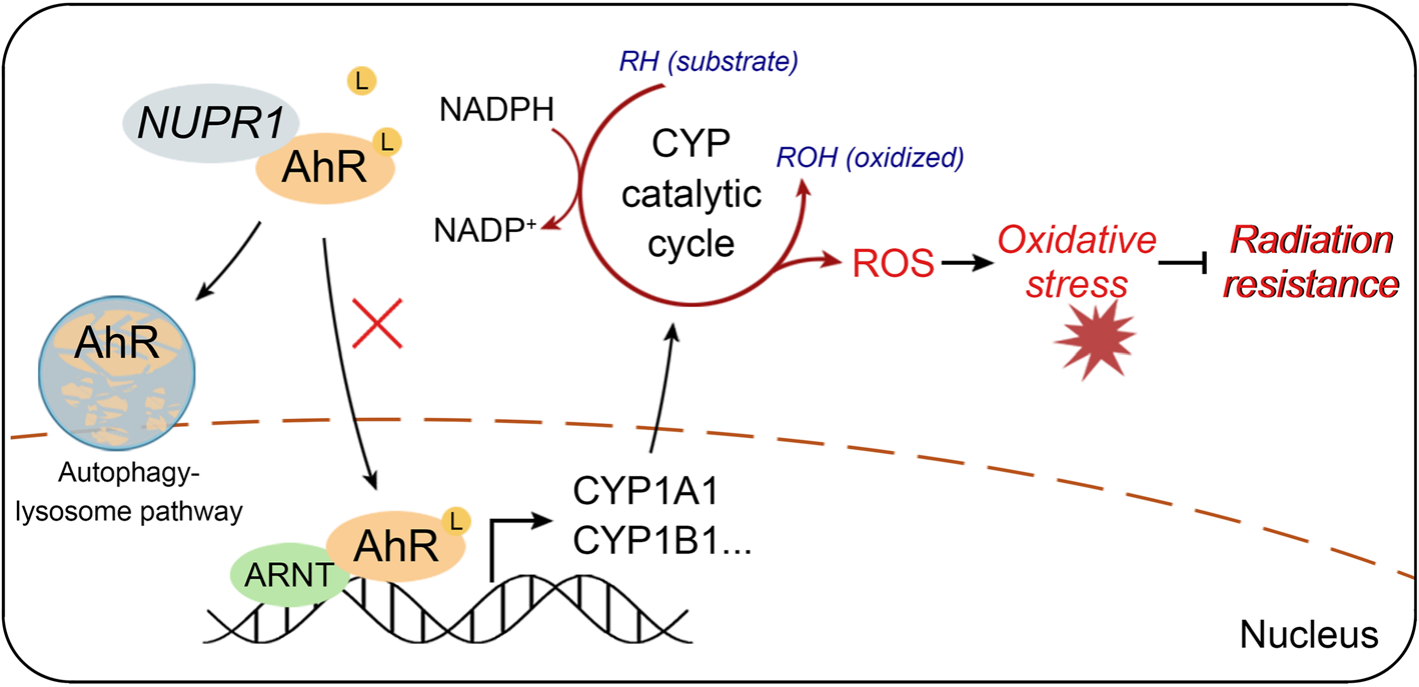
A schematic model of the NUPR1/AhR/CYP signal axis showing it promotes radiation resistance of HCC

In summary, our study attempted to validate the radioresistant role of NUPR1 in HCC. Our findings provide new insights for understanding the underlying mechanism of NUPR1, that is to attenuate CYPs-mediated ROS formation and oxidative stress by complexing with and downregulating AhR, therefore promoting radioresistance of HCC. Clinical data suggested that NUPR1 and AhR could be predictive biomarkers for the RT response of HCC patients. Specific inhibiting NUPR1 by ZZW-115 significantly improved the vulnerability of HCC to IR in the xenograft mice model. All the results implicated that NUPR1/AhR/CYP signaling axis might serve as the potential target for improving radiotherapeutic efficacy in HCC (Fig. 8).

## Supplementary Information

**Additional file 1: Figure S1.** NUPR1 promotes tumor growth and radiation resistance of HCC. **Figure S2.** NUPR1 suppresses IR-induced apoptosis and lipid peroxidation. **Figure S3.** NAC attenuates oxidative stress induced by NUPR1 silencing upon IR. **Figure S4.** CYP inhibitor alizarin impedes ROS generation and oxidative stress upon IR exposure. **Figure S5.** NUPR1 modulates the protein levels and nuclear translocation of AhR. **Figure S6.** NUPR1 interacts with AhR and promotes degradation via the autophagy-lysosome pathway. **Figure S7.** NUPR1 inhibits oxidative stress via AhR/CYP signaling. **Figure S8.** NUPR1 is upregulated in HCC tissues and correlates with glutathione metabolism. **Table S1.** List of NUPR1 shRNA and siRNA coding sequences. **Table S2.** List of primers used in this study.

**Additional file 2.** The images of the original, uncropped gels/blots.

## Data Availability

Data are available upon reasonable request from corresponding authors, Yi Ding (dingyi197980@126.com), Li Liang (lli@smu.edu.cn), and Guoxin Li (gzliguoxin@163.com).

## Abbreviations

AhR: Aryl hydrocarbon receptor
ARNT: Aryl hydrocarbon receptor nuclear translocator
CQ: Chloroquine
CYP: Cytochrome P450
GSEA: Gene-set enrichment analysis
HCC: Hepatocellular carcinoma
IR: Ionizing radiation
NAC: N-acetyl-L-cysteine
NADPH: Nicotinamide adenine dinucleotide phosphate
NUPR1: Nuclear protein 1
OS: Overall survival
qRT-PCR: Quantitative real-time polymerase chain reaction
ROS: Reactive oxygen species
RT: Radiation therapy
TCGA: The Cancer Genome Atlas
