# Supplementary material for: NUPR1 contributes to radiation resistance by maintaining ROS homeostasis via AhR/CYP signal axis in hepatocellular carcinoma
Source: BMC Med. 2022 Oct 19;20:365. doi: 10.1186/s12916-022-02554-3 (PMC9580158; doi:10.1186/s12916-022-02554-3)
Supplement: Supplementary file 1 — Additional file 1: Figure S1. NUPR1 promotes tumor growth and radiation resistance of HCC. Figure S2. NUPR1 suppresses IR-induced apoptosis and lipid peroxidation. Figure S3. NAC attenuates oxidative stress induced by NUPR1 silencing upon IR. Figure S4. CYP inhibitor alizarin impedes ROS generation and oxidative stress upon IR exposure. Figure S5. NUPR1 modulates the protein levels and nuclear translocation of AhR. Figure S6. NUPR1 interacts with AhR and promotes degradation via the autophagy-lysosome pathway. Figure S7. NUPR1 inhibits oxidative stress via AhR/CYP signaling. Figure S8. NUPR1 is upregulated in HCC tissues and correlates with glutathione metabolism. Table S1. List of NUPR1 shRNA and siRNA coding sequences. Table S2. List of primers used in this study. [file 12916_2022_2554_MOESM1_ESM.docx]

Supplementary information for:

**NUPR1 Contributes to Radiation Resistance by Maintaining ROS Homeostasis via AhR/CYP Signal Axis in Hepatocellular Carcinoma**

**Supplementary Figures and Tables:**

**Fig. S1** NUPR1 promotes tumor growth and radiation resistance of HCC.

**Fig. S2** NUPR1 suppresses IR-induced apoptosis and lipid peroxidation.

**Fig. S3** NAC attenuates oxidative stress induced by NUPR1 silencing upon IR.

**Fig. S4** CYP inhibitor alizarin impedes ROS generation and oxidative stress upon IR exposure.

**Fig. S5** NUPR1 modulates the protein levels and nuclear translocation of AhR.

**Fig. S6** NUPR1 interacts with AhR and promotes degradation via the autophagy-lysosome pathway.

**Fig. S7** NUPR1 inhibits oxidative stress via AhR/CYP signaling.

**Fig. S8** NUPR1 is upregulated in HCC tissues and correlates with glutathione metabolism.

**Table S1** List of NUPR1 shRNA and siRNA coding sequences.

**Table S2** List of primers used in this study.

**Supplementary figures:**

**
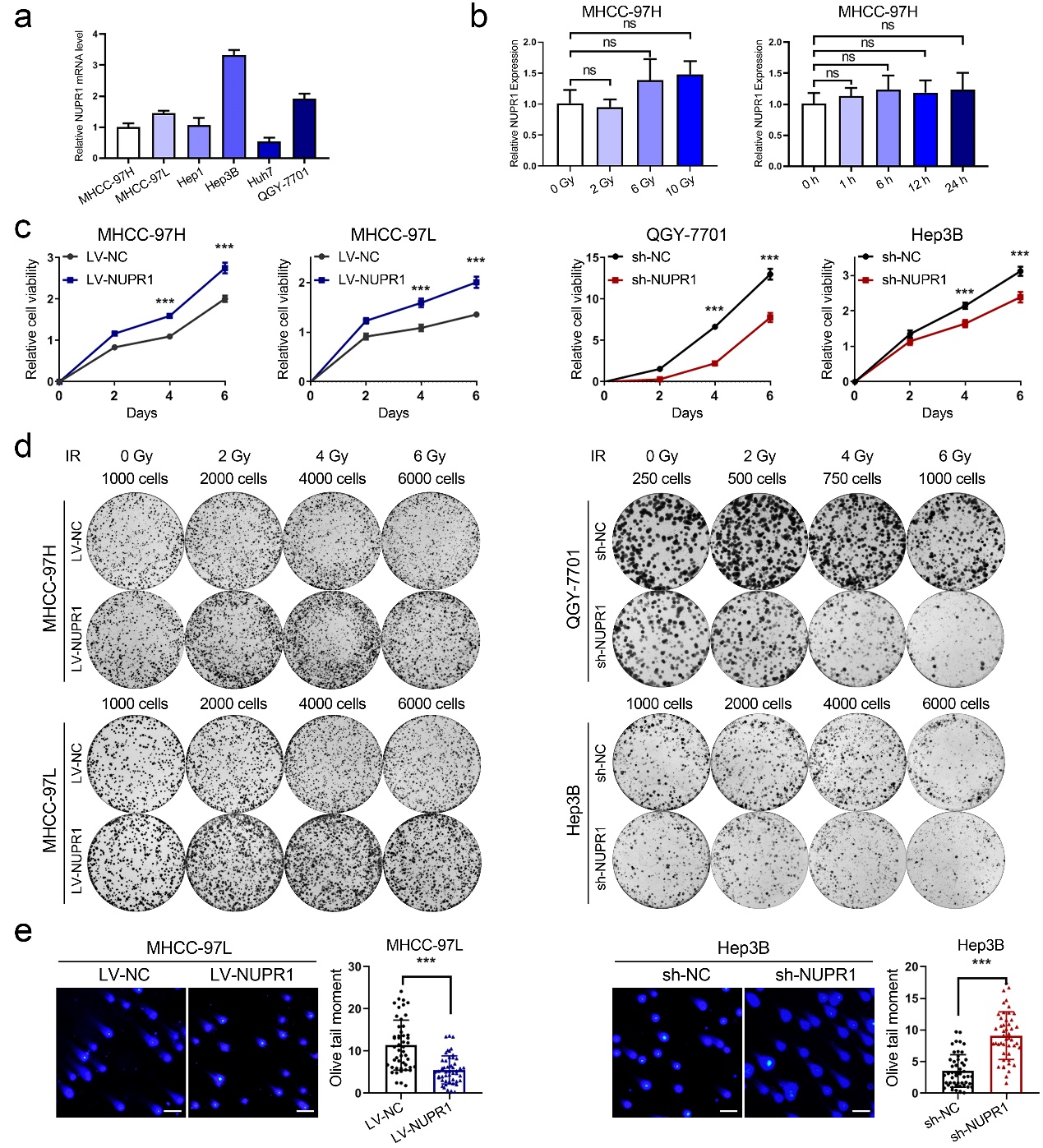
Figure S1. NUPR1 promotes tumor growth and radiation resistance of HCC.**

**a** The mRNA levels of NUPR1 in a panel of HCC cell lines were measured by qRT-PCR. Individual RNA values were normalized to β-actin values. **b** The mRNA expression of NUPR1 was evaluated in MHCC-97H cells after a different dose of IR treatment (0, 2, 6, 10 Gy) at different timing by qRT-PCR. Individual RNA values were normalized to β-actin values. **c** The cell proliferation of stably transfected NUPR1 overexpression or knockdown cell lines was monitored with CCK8 assays. **d** Colony formation images of MHCC-97H/MHCC-97L cells with NUPR1 overexpression and QGY-7701/Hep3B cells with NUPR1 knockdown were shown after a different dose of IR treatment (0, 2, 4, 6 Gy). **e** DNA double-strand breaks of NUPR1-overexpressing MHCC-97L and NUPR1-knockdown Hep3B cells were detected by comet assays at 24 h after exposure to IR (8 Gy) (left, representative images, scale bar: 50 µm; right, bar graphs indicating the average tail moment per cell). Data are the mean of biological triplicates and are shown as the mean ± SD. *P* values: **P* < 0.05; ***P* < 0.01; ****P* < 0.001 by two-tailed Student’s *t*-test.

**
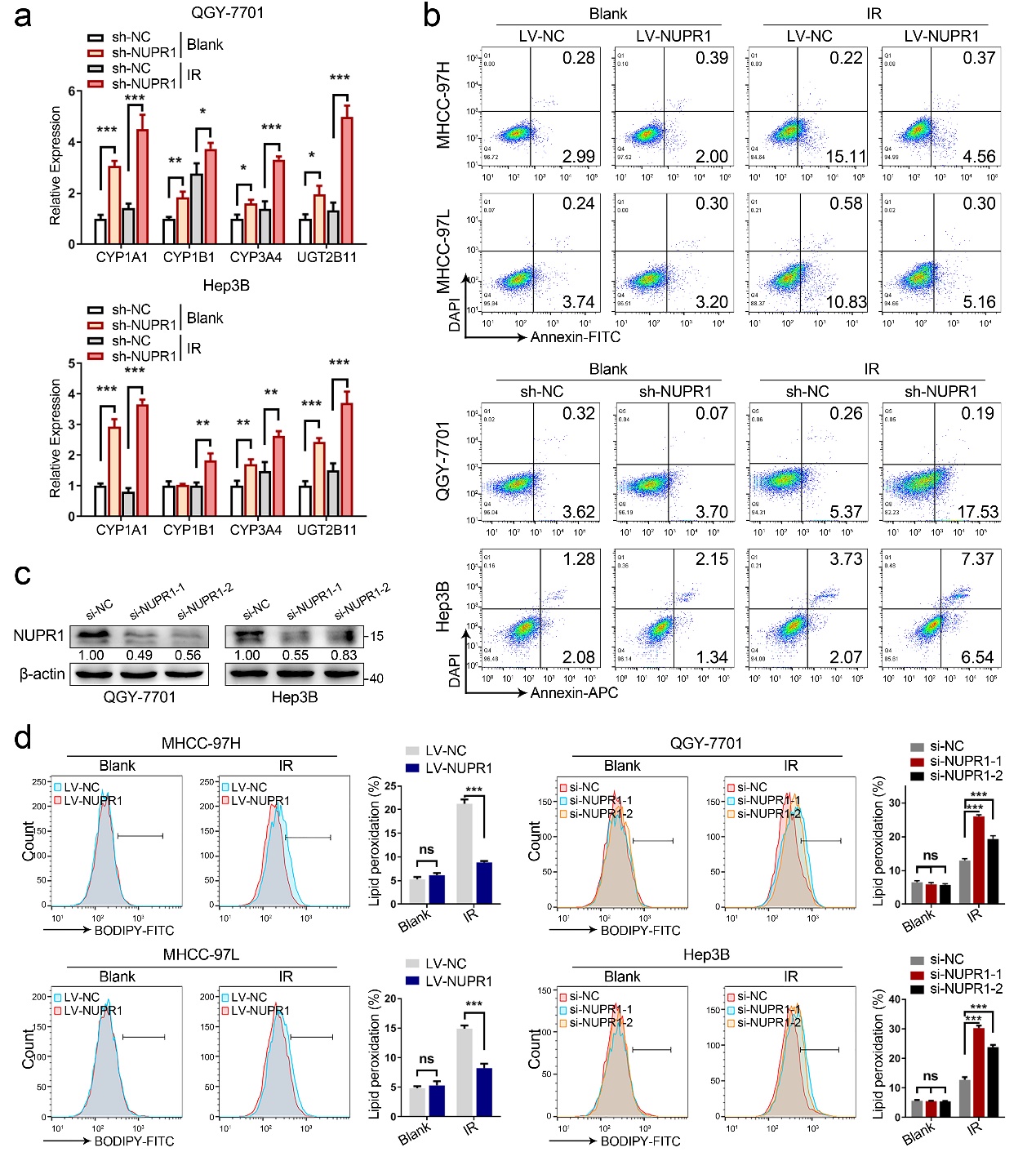
Figure S2. NUPR1 suppresses IR-induced apoptosis and lipid peroxidation.**

**a** The mRNA levels of CYP enzymes in QGY-7701/Hep3B cells with NUPR1 knockdown were analyzed by qRT-PCR. Individual RNA values were normalized to β-actin values. Data are mean ± SD from three replicates. **b** Apoptotic cells were quantified by annexin V-APC/DAPI or annexin V-FITC/DAPI double staining in indicated cell lines using a flow cytometer. **c** The protein levels of NUPR1 in QGY-7701/Hep3B cells transfected with siRNA against NUPR1 or negative control were verified by western blot. **d** Lipid peroxidation was assessed in stable NUPR1-overexpressing or knockdown cell lines after exposure to 8 Gy of IR. Bar graphs show the relative lipid peroxidation levels by C11-BODIPY staining in the indicated cells. Data are the mean of biological triplicates and are shown as the mean ± SD. *P* values: **P* < 0.05; ***P* < 0.01; ****P* < 0.001 and ns, not significant by Student’s *t-*test.

**
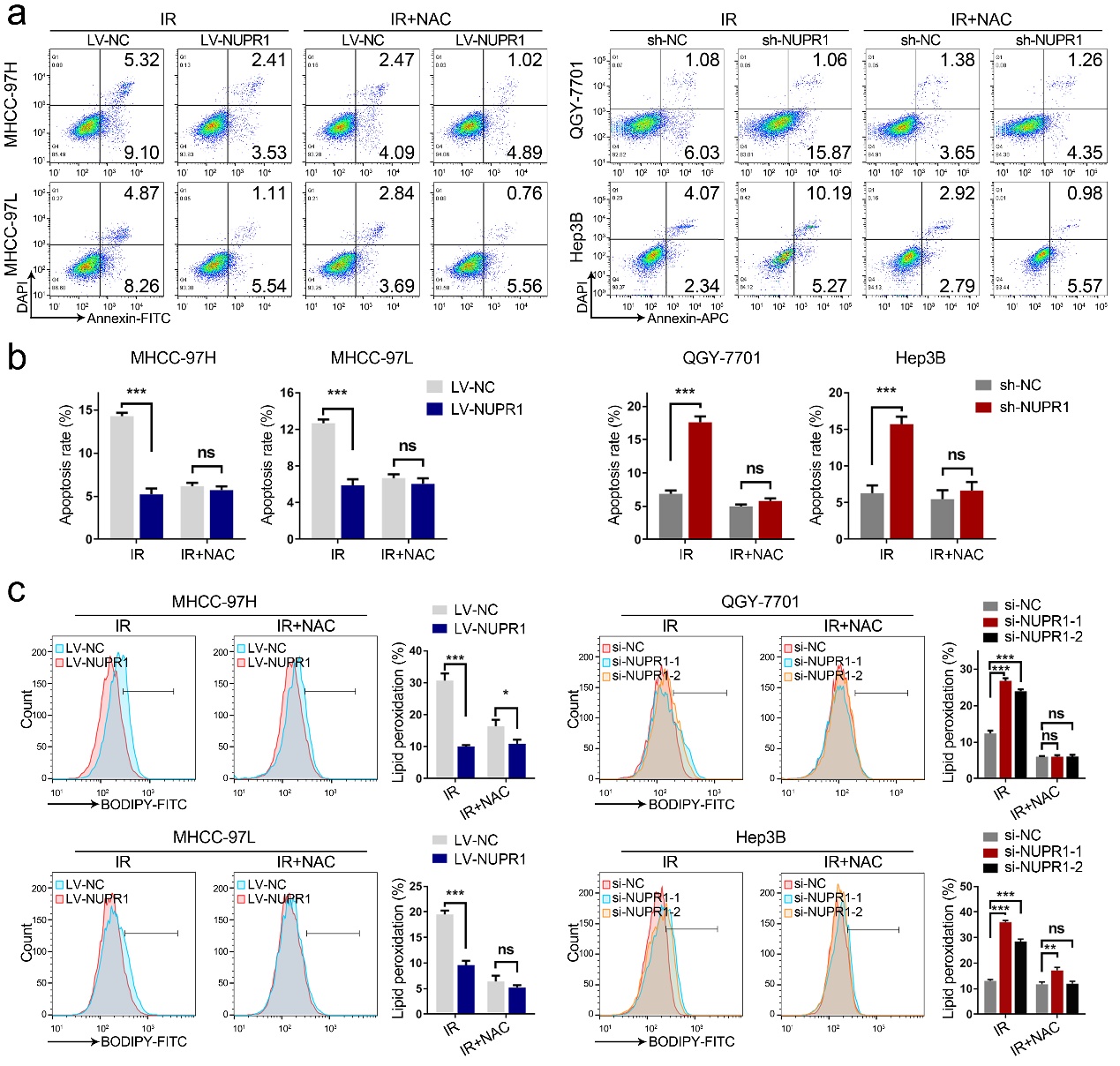
Figure S3. NAC attenuates oxidative stress induced by NUPR1 silencing upon IR.**

**a** Representative flow cytometry images of cell apoptosis pretreated with or without NAC are shown after IR (8 Gy) exposure are shown. **b** Bar graphs show the relative levels of apoptosis in the indicated cell lines**. c** Lipid peroxidation assessment was performed in cell lines with a different NUPR1 expression status pretreated with 5mM NAC for 24 h followed by exposure to 8 Gy of IR. Bar graphs show the IR-induced relative levels of lipid peroxidation by C11-BODIPY staining in the indicated cells. Data are the mean of biological triplicates and are shown as the mean ± SD. *P* values: **P* < 0.05; ***P* < 0.01; ****P* < 0.001 and ns, not significant by Student’s *t-*test.

**
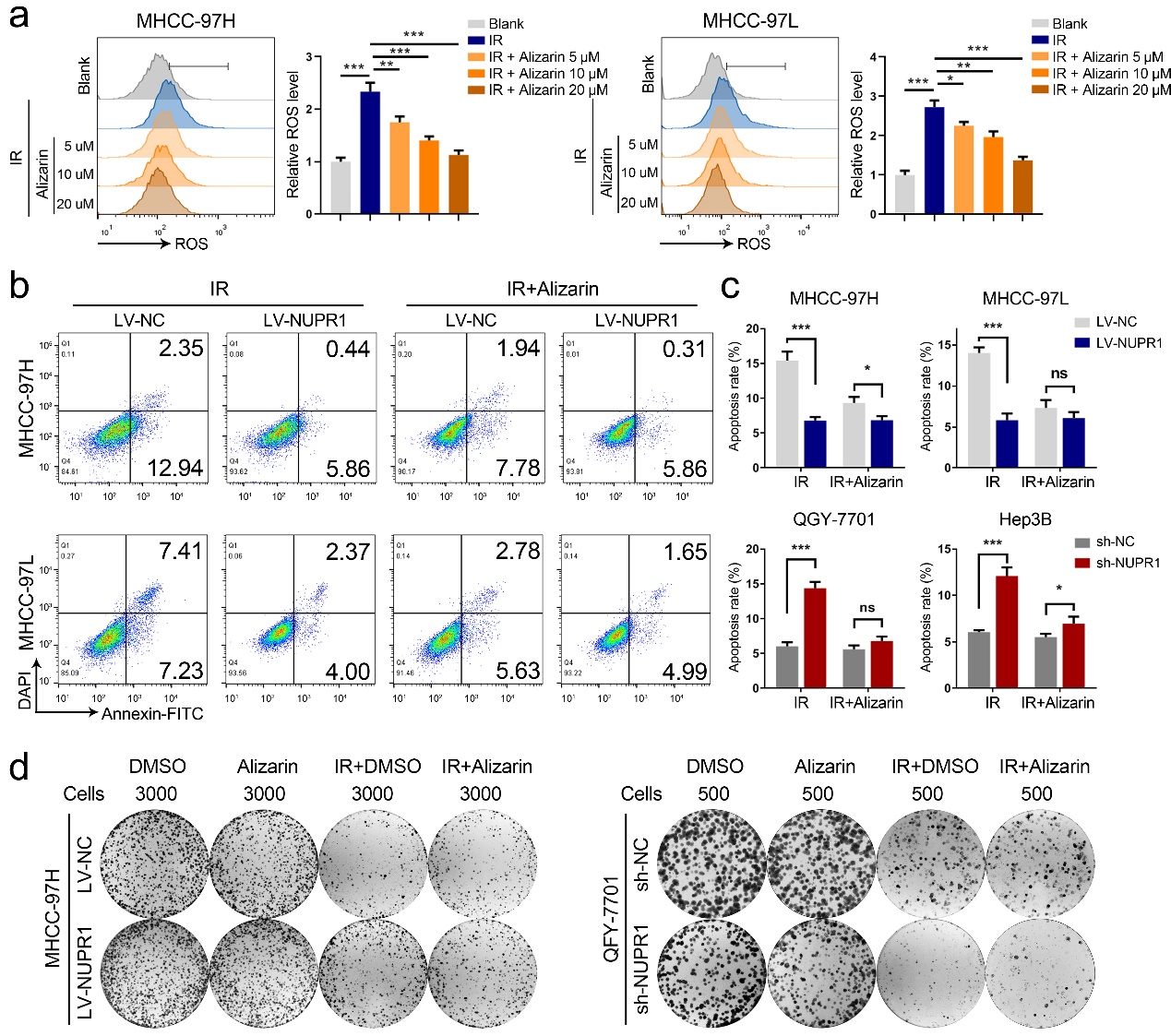
Figure S4. CYP inhibitor alizarin impedes ROS generation and oxidative stress upon IR exposure.**

**a** ROS levels were determined in MHCC-97H and MHCC-97L cells pretreated with a concentration gradient of alizarin (5, 10, 20 μM), followed by exposure to 8 Gy of IR. **b** Representative flow cytometry analyses of apoptosis treated with or without alizarin after IR (8 Gy) are shown on left. **c** Bar graphs show the relative levels of apoptosis in the indicated cell lines**. d** Colony formation images of NUPR1-overexpressing MHCC-97H (3000 cells) and QGY-770 with NUPR1 knockdown (500 cells) were shown after IR (6 Gy) combined with or without 20 μM alizarin. Data are the mean of biological triplicates and are shown as the mean ± SD. *P* values: **P* < 0.05; ***P* < 0.01; ****P* < 0.001 and ns, not significant by two-tailed Student’s *t-*test.

**
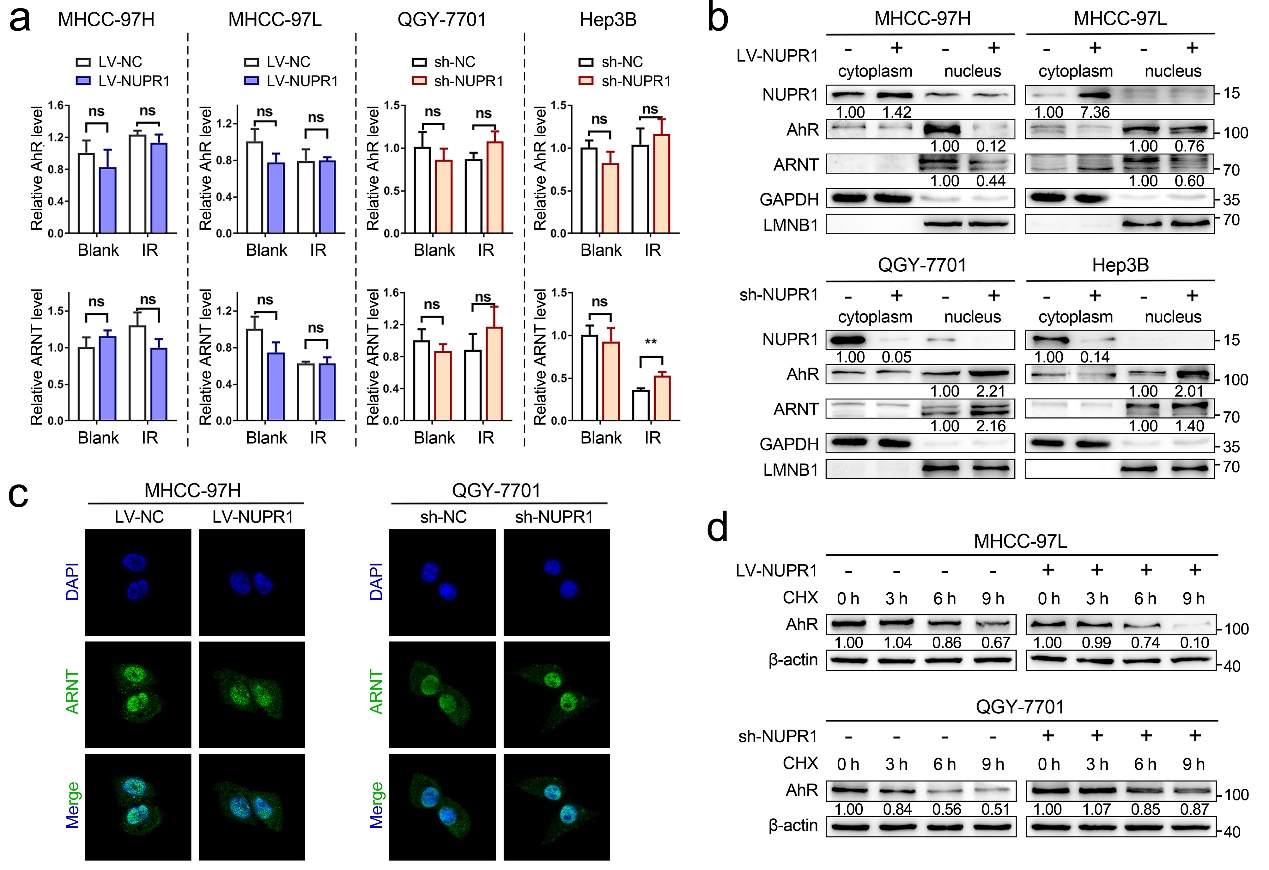
Figure S5. NUPR1 modulates the protein levels and nuclear translocation of AhR.**

**a** The mRNA levels of AhR and ARNT showed no significant changes in stably transfected NUPR1 overexpressing or knockdown cell lines by qRT-PCR. Data are mean ± SD from three replicates. **b** The expression levels of AhR and ARNT in lysates from cellular cytoplasm and nucleus were determined by western blot. LMNB1 is a nuclear marker, and GAPDH was loaded as a cytoplasmic marker. **c** Representative immunofluorescence images show the location of ARNT (green) and nuclei counterstained with DAPI (blue) in LV-NC/LV-NUPR1 MHCC-97H and sh-NC/sh-NUPR1 QGY-7701 cells. **d** The levels of AhR protein in cell lysates collected at different timing with 20 µg/mL CHX treatment were determined by western blot. Data are the mean of biological triplicates and are shown as the mean ± SD. *P* values: ***P* < 0.01 and ns, not significant.


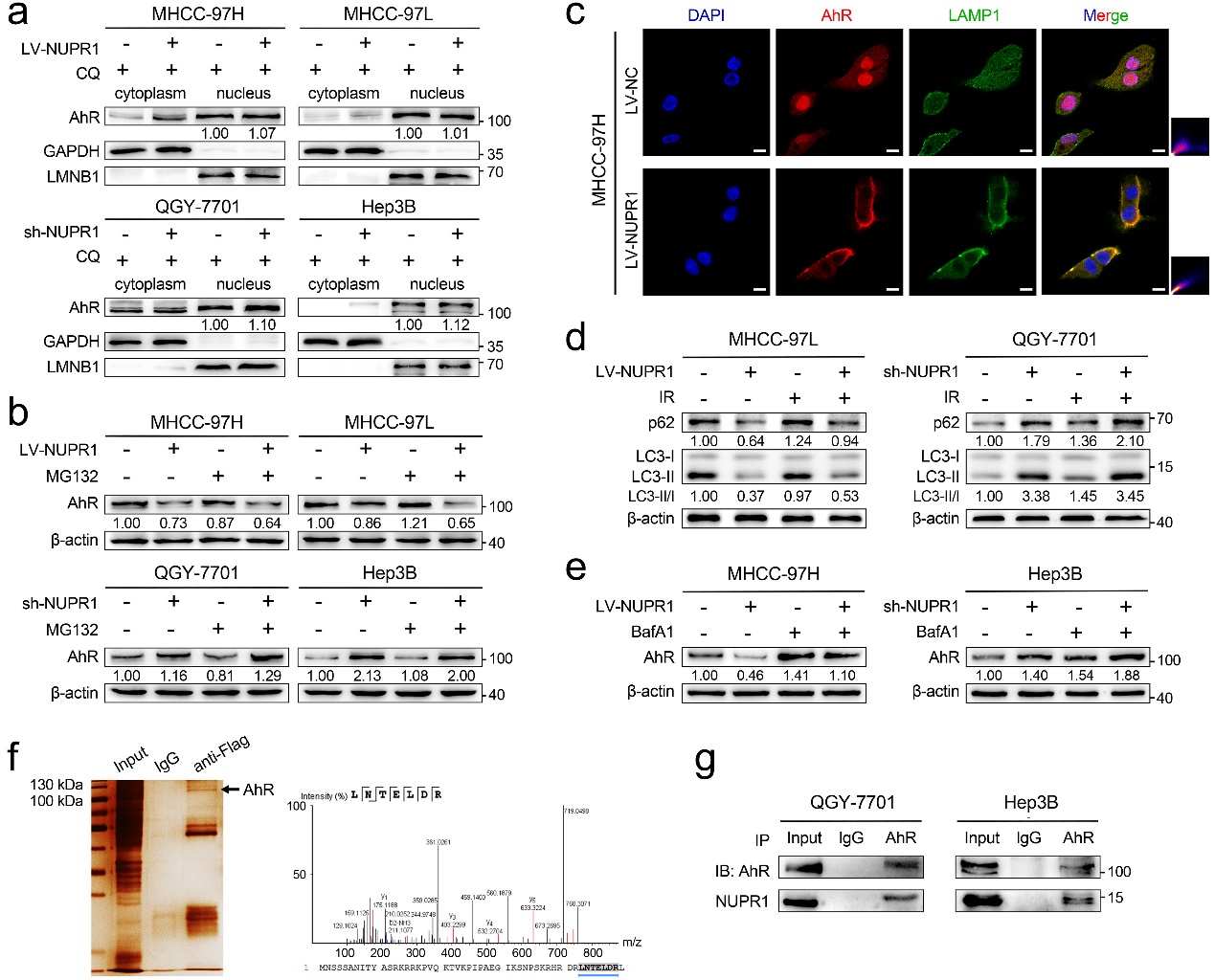


**Figure S6. NUPR1 interacts with AhR and promotes degradation via the autophagy-lysosome pathway.**

**a** The expression levels of AhR in lysates from cellular cytoplasm and nucleus pretreated with 20 μM CQ were determined by western blot. LMNB1 is a nuclear marker, and GAPDH was loaded as a cytoplasmic marker. **b** Western blot was used to verify the AhR levels in indicated cell lysates with or without 10 µM MG132 treatment. **c** Representative immunofluorescence images show the distribution of AhR (red), LAMP1 (green), and DAPI (blue) in LV-NC and LV-NUPR1 MHCC-97H cells. Scale bar: 10 µm. **d** The levels of LC3-II and p62 in cells with a different NUPR1 expression status were determined by western blot. **e** Western blot of AhR expression in indicated cell lysates pretreated with or without 50 nM BafA1. **f** Silver staining of anti-Flag products was presented on left. LC-MS/MS analysis was used to determine AhR as one of the NUPR1-interacting proteins (right). **g** Western blot analysis of NUPR1 and AhR in products immunoprecipitated with AhR antibodies or IgG in QGY-7701 and Hep3B cells.


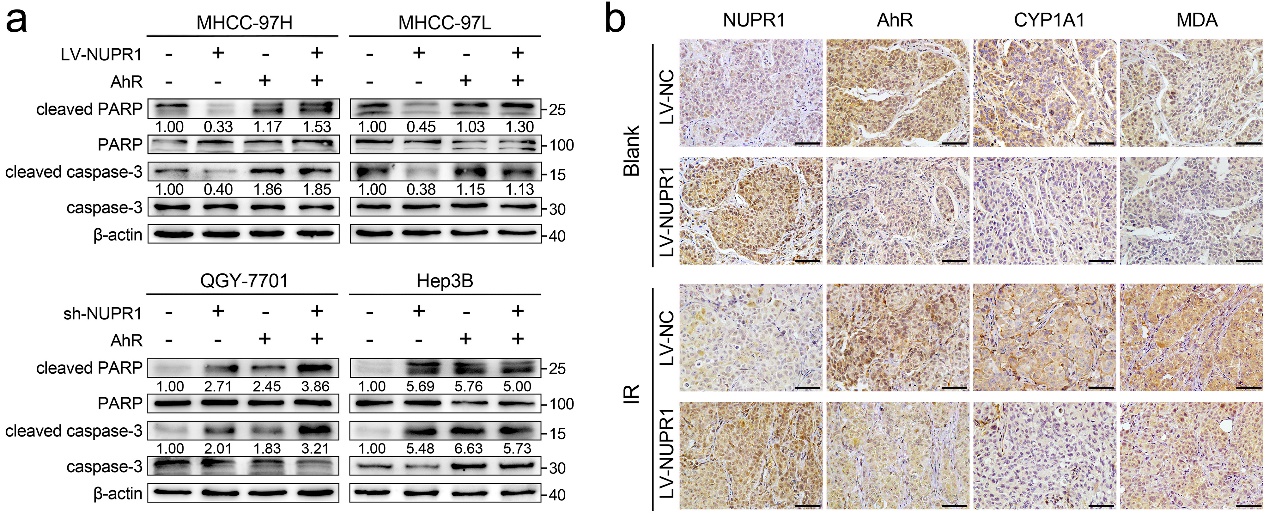


**Figure S7. NUPR1 inhibits oxidative stress via AhR/CYP signaling.**

**a** Western blot of the protein levels of total/cleaved caspase-3 and total/cleaved PARP was performed in indicated cells with AhR overexpression or empty vector under 8 Gy treatment. **b** Representative IHC images of NUPR1, AhR, CYP1A1, and malondialdehyde (MDA) expression in xenograft tumors derived from MHCC-97H with or without stable NUPR1 overexpression are shown. Scale bar: 50 µm.

**
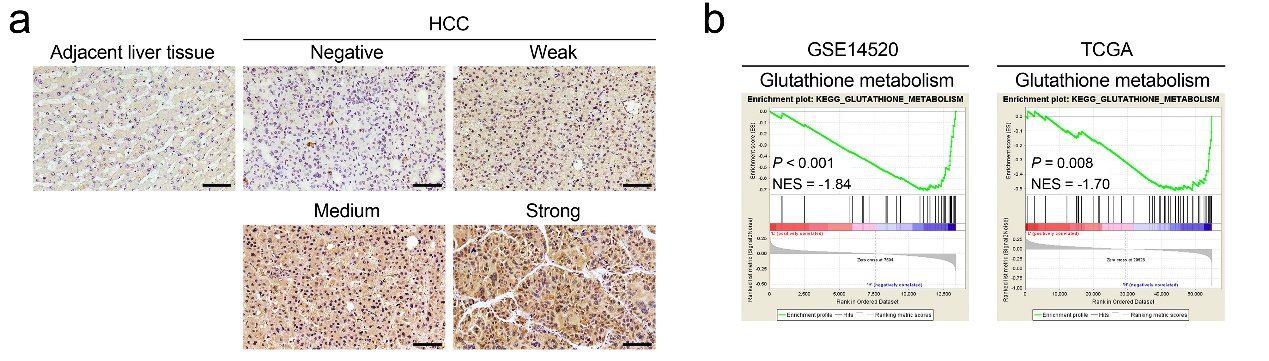
**

**Figure S8. NUPR1 is upregulated in HCC tissues and correlates with glutathione metabolism.**

**a** Representative IHC images show the expression level of NUPR1 in HCC tissue and corresponding adjacent liver tissue. Scale bar: 50 µm. **b** GSEA of HCC samples from TCGA and GSE14520 datasets showed that the glutathione metabolism pathway was positively correlated with NUPR1 expression.

**Table S1. List of NUPR1 shRNA and siRNA coding sequences**

| **Name** | **Target sequence** |
| --- | --- |
| sh-NUPR1#1 | GATCCGAGAGGAAACTGGTGACCAAGTTCAAGAGACTTGGTCACCAGTTTCCTCTCTTTTTTA |
| sh-NUPR1#2 | GATCCGTGGACACTACACCCAGCAATATTCAAGAGATATTGCTGGGTGTAGTGTCCATTTTTTA |
| sh-NC | TTCTCCGAACGTGTCACGTTTCAAGAGAACGTGACACGTTCGGAGAATTTTTT |
| si-NUPR1#1 | GAGAGGAAACUGGUGACCAAGTT |
| si-NUPR1#2 | CCUGAGCAAUAGAGUGAAATT |
| si-NC | UUCUCCGAACGUGUCACGUTT |

**Table S2. List of primers used in this study**

| **Name** | **Forward** | **Reverse** |
| --- | --- | --- |
| **Gene** |  |  |
| NUPR1 | GACTGAGTCTCTGAGGGGCTAC | GTTGCTGCCACCCTGGAGGA |
| AhR | CACTCAGACTACCACACATCTT | AGGAATCCACTGGATGTCAAAT |
| ARNT | CTGCCAACCCCGAAATGACAT | CGCCGCTTAATAGCCCTCTG |
| CYP1A1 | CGTTGTGTCTTTGTAAACCAGT | ACTTAACACCTTGTCGATAGCA |
| CYP1B1 | GAACTTTGATCCAGCTCGATTC | TTAGAAAGTTCTTCGCCAATGC |
| CYP3A4 | CACGAGCAGTGTTCTCTCCTT | CACAGTATCATAGGTGGGTGGT |
| UGT2B11 | TCTCTGGGGTCAGTGATAAGTA | CGAGTATTGAGACCTAAGGCAT |
| β-actin | ATCCATGTGACCATGAGGAAATG | TCGGCTAGTTAGGGTACACTTC |
