# Supplementary material for: NUPR1 contributes to radiation resistance by maintaining ROS homeostasis via AhR/CYP signal axis in hepatocellular carcinoma
Source: BMC Med. 2022 Oct 19;20:365. doi: 10.1186/s12916-022-02554-3 (PMC9580158; doi:10.1186/s12916-022-02554-3)
Supplement: Supplementary file 2 — Additional file 2. The images of the original, uncropped gels/blots. [file 12916_2022_2554_MOESM2_ESM.docx]

NUPR1 in Fig. 1a


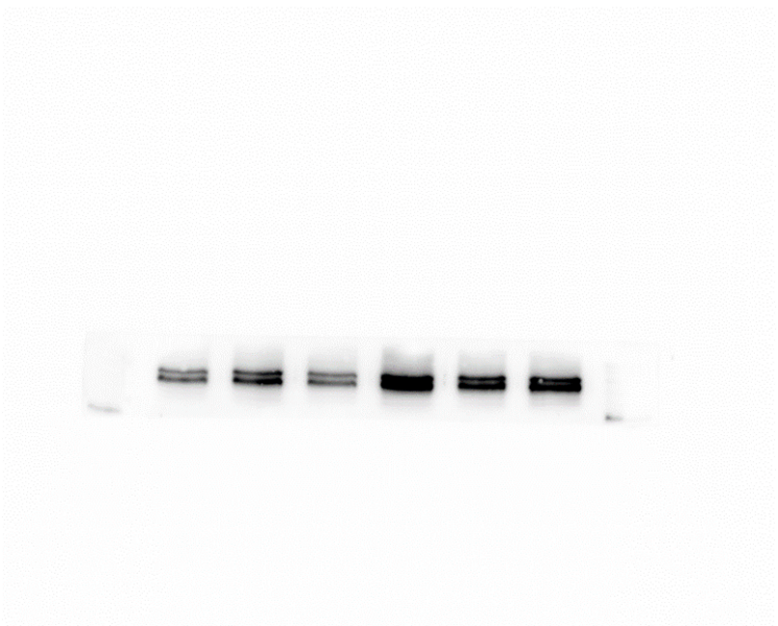


β-actin in Fig. 1a





NUPR1 in Fig. 1b

MHCC-97H





MHCC-97L






QGY-7701

Hep3B





β-actin in Fig. 1b

MHCC-97H





MHCC-97L





QGY-7701





Hep3B





γH2AX in Fig. 1f

MHCC-97H





MHCC-97L





QGY-7701





Hep3B





β-actin in Fig. 1f

MHCC-97H





MHCC-97L





QGY-7701





Hep3B





CYP1A1 in Fig. 3c

MHCC-97H





MHCC-97L





QGY-7701





Hep3B





CYP1B1 in Fig. 3c

MHCC-97H





MHCC-97L





QGY-7701





Hep3B





CYP3A4 in Fig. 3c

MHCC-97H





MHCC-97L





QGY-7701





Hep3B





β-actin in Fig. 3c

MHCC-97H





MHCC-97L





QGY-7701





Hep3B





cleaved PARP in Fig. 3g

MHCC-97H





MHCC-97L





QGY-7701





Hep3B





PARP in Fig. 3g

MHCC-97H





MHCC-97L





QGY-7701





Hep3B





cleaved caspase-3 in Fig. 3g

MHCC-97H





MHCC-97L





QGY-7701





Hep3B





caspase-3 in Fig. 3g

MHCC-97H





MHCC-97L





QGY-7701





Hep3B





β-actin in Fig. 3g

MHCC-97H





MHCC-97L





QGY-7701





Hep3B





cleaved PARP in Fig. 4d

MHCC-97H





MHCC-97L





QGY-7701





Hep3B





PARP in Fig. 4d

MHCC-97H





MHCC-97L





QGY-7701





Hep3B





cleaved caspase-3 in Fig. 4d

MHCC-97H





MHCC-97L





QGY-7701





Hep3B





caspase-3 in Fig. 4d

MHCC-97H





MHCC-97L





QGY-7701





Hep3B





β-actin in Fig. 4d

MHCC-97H





MHCC-97L





QGY-7701





Hep3B





AhR in Fig. 5a

MHCC-97H





MHCC-97L





QGY-7701





Hep3B





ARNT in Fig. 5a

MHCC-97H





MHCC-97L





QGY-7701





Hep3B





HSP90 in Fig. 5a

MHCC-97H





MHCC-97L





QGY-7701





Hep3B





β-actin in Fig. 5a

MHCC-97H





MHCC-97L





QGY-7701





Hep3B





AhR in Fig. 5c

MHCC-97H LV-NC





MHCC-97H LV-NUPR1





Hep3B sh-NC





Hep3B sh-NUPR1





β-actin in Fig. 5c

MHCC-97H LV-NC





MHCC-97H LV-NUPR1





Hep3B sh-NC





Hep3B sh-NUPR1





AhR in Fig. 5d

MHCC-97H





MHCC-97L





QGY-7701

Hep3B

β-actin in Fig. 5d

MHCC-97H

MHCC-97L

QGY-7701

Hep3B

AhR in Fig. 5g

MHCC-97H LV-NUPR1

MHCC-97L LV-NUPR1

Flag in Fig. 5g

MHCC-97H LV-NUPR1

MHCC-97L LV-NUPR1

AhR in Fig. 5h

MHCC-97H LV-NUPR1

MHCC-97L LV-NUPR1

NUPR1 in Fig. 5h

MHCC-97H LV-NUPR1

MHCC-97L LV-NUPR1

p62 in Fig. 5f

MHCC-97H

Hep3B

LC3 in Fig. 5f

MHCC-97H

Hep3B

β-actin in Fig. 5f

MHCC-97H

Hep3B

AhR in Fig. 6a

MHCC-97H

MHCC-97L

QGY-7701

Hep3B

CYP1A1 in Fig. 6a

MHCC-97H

MHCC-97L

QGY-7701

Hep3B

CYP1B1 in Fig. 6a

MHCC-97H

MHCC-97L

QGY-7701

Hep3B

β-actin in Fig. 6a

MHCC-97H

MHCC-97L

QGY-7701

Hep3B

NUPR1 in Fig. S2c

QGY-7701

Hep3B

β-actin in Fig. S2c

QGY-7701

Hep3B

NUPR1 in Fig. S5b

MHCC-97H

MHCC-97L

QGY-7701

Hep3B

AhR in Fig. S5b

MHCC-97H

MHCC-97L

QGY-7701

Hep3B

ARNT in Fig. S5b

MHCC-97H

MHCC-97L

QGY-7701

Hep3B

GAPDH in Fig. S5b

MHCC-97H

MHCC-97L

QGY-7701

Hep3B

LMNB1 in Fig. S5b

MHCC-97H

MHCC-97L

QGY-7701

Hep3B

AhR in Fig. S5d

MHCC-97L LV-NC

MHCC-97L LV-NUPR1

QGY-7701 sh-NC

QGY-7701 sh-NUPR1

β-actin in Fig. S5d

MHCC-97L LV-NC

MHCC-97L LV-NUPR1

QGY-7701 sh-NC

QGY-7701 sh-NUPR1

AhR in Fig. S6a

MHCC-97H

MHCC-97L

QGY-7701

Hep3B

GAPDH in Fig. S6a

MHCC-97H

MHCC-97L

QGY-7701

Hep3B

LMNB1 in Fig. S6a

MHCC-97H

MHCC-97L

QGY-7701

Hep3B

AhR in Fig. S6b

MHCC-97H

MHCC-97L

QGY-7701

Hep3B

β-actin in Fig. S6b

MHCC-97H

MHCC-97L

QGY-7701

Hep3B

p62 in Fig. S6d

MHCC-97L

QGY-7701

LC3 in Fig. S6d

MHCC-97L

QGY-7701

β-actin in Fig. S6d

MHCC-97L

QGY-7701

AhR in Fig. S6e

MHCC-97H

Hep3B

β-actin in Fig. S6e

MHCC-97H

Hep3B

AhR in Fig. S6g

QGY-7701

Hep3B

NUPR1 in Fig. S6g

QGY-7701

Hep3B

cleaved PARP in Fig. S7a

MHCC-97H

MHCC-97L

QGY-7701

Hep3B

PARP in Fig. S7a

MHCC-97H

MHCC-97L

QGY-7701

Hep3B

cleaved caspase-3 in Fig. S7a

MHCC-97H

MHCC-97L

QGY-7701

Hep3B

caspase-3 in Fig. S7a

MHCC-97H

MHCC-97L

QGY-7701

Hep3B

β-actin in Fig. S7a

MHCC-97H

MHCC-97L

QGY-7701

Hep3B
